# Alternatively activated macrophages promote resolution of necrosis following acute liver injury

**DOI:** 10.1016/j.jhep.2020.02.031

**Published:** 2020-08

**Authors:** Philip Starkey Lewis, Lara Campana, Niya Aleksieva, Jennifer Ann Cartwright, Alison Mackinnon, Eoghan O'Duibhir, Timothy Kendall, Matthieu Vermeren, Adrian Thomson, Victoria Gadd, Benjamin Dwyer, Rhona Aird, Tak-Yung Man, Adriano Giorgio Rossi, Lesley Forrester, B. Kevin Park, Stuart John Forbes

**Affiliations:** 1 MRC Centre for Regenerative Medicine, University of Edinburgh, 5 Little France Drive, Edinburgh, United Kingdom; 2 Centre for Inflammation Research, Queen's Medical Research Institute, University of Edinburgh, Edinburgh, United Kingdom; 3 Edinburgh Pathology, University of Edinburgh, Edinburgh, United Kingdom; 4 UK Regenerative Medicine Platform Safety and Efficacy Hub, University of Liverpool, Liverpool, United Kingdom; 5 Edinburgh Preclinical Imaging, BHF Centre for Cardiovascular Science, University of Edinburgh, Edinburgh, United Kingdom; 6 MRC Centre for Drug Safety Science, University of Liverpool, Ashton Street, Liverpool, United Kingdom

**Keywords:** Acetaminophen, Liver regeneration, Macrophages, Necrosis, Phagocytosis

## Abstract

**Background & Aim:**

Following acetaminophen (APAP) overdose, acute liver injury (ALI) can occur in patients that present too late for N-acetylcysteine treatment, potentially leading to acute liver failure, systemic inflammation, and death. Macrophages influence the progression and resolution of ALI due to their innate immunological function and paracrine activity. Syngeneic primary bone marrow-derived macrophages (BMDMs) were tested as a cell-based therapy in a mouse model of APAP-induced ALI (APAP-ALI).

**Methods:**

Several phenotypically distinct BMDM populations were delivered intravenously to APAP-ALI mice when hepatic necrosis was established, and then evaluated based on their effects on injury, inflammation, immunity, and regeneration. *In vivo* phagocytosis assays were used to interrogate the phenotype and function of alternatively activated BMDMs (AAMs) post-injection. Finally, primary human AAMs sourced from healthy volunteers were evaluated in immunocompetent APAP-ALI mice.

**Results:**

BMDMs rapidly localised to the liver and spleen within 4 h of administration. Injection of AAMs specifically reduced hepatocellular necrosis, HMGB1 translocation, and infiltrating neutrophils following APAP-ALI. AAM delivery also stimulated proliferation in hepatocytes and endothelium, and reduced levels of several circulating proinflammatory cytokines within 24 h. AAMs displayed a high phagocytic activity both *in vitro* and in injured liver tissue post-injection. Crosstalk with the host innate immune system was demonstrated by reduced infiltrating host Ly6C^hi^ macrophages in AAM-treated mice. Importantly, therapeutic efficacy was partially recapitulated using clinical-grade primary human AAMs in immunocompetent APAP-ALI mice, underscoring the translational potential of these findings.

**Conclusion:**

We identify that AAMs have value as a cell-based therapy in an experimental model of APAP-ALI. Human AAMs warrant further evaluation as a potential cell-based therapy for APAP overdose patients with established liver injury.

**Lay summary:**

After an overdose of acetaminophen (paracetamol), some patients present to hospital too late for the current antidote (N-acetylcysteine) to be effective. We tested whether macrophages, an injury-responsive leukocyte that can scavenge dead/dying cells, could serve as a cell-based therapy in an experimental model of acetaminophen overdose. Injection of alternatively activated macrophages rapidly reduced liver injury and reduced several mediators of inflammation. Macrophages show promise to serve as a potential cell-based therapy for acute liver injury.

## Highlights

• Primary BMDMs localised to liver and spleen within hours following intravenous injection in mice.
• AAMs were highly phagocytic and i.v. transfer elicited reductions in necrotic area, HMGB1 translocation, and hepatic neutrophil infiltration.
• AAM injection reduced inflammatory mediators and stimulated hepatocyte/endothelium proliferation in injured liver.
• Injection of clinical-grade human AAMs could partially recapitulate the efficacy of murine AAMs in immunocompetent mice.

## Graphical abstract

**Figure:**
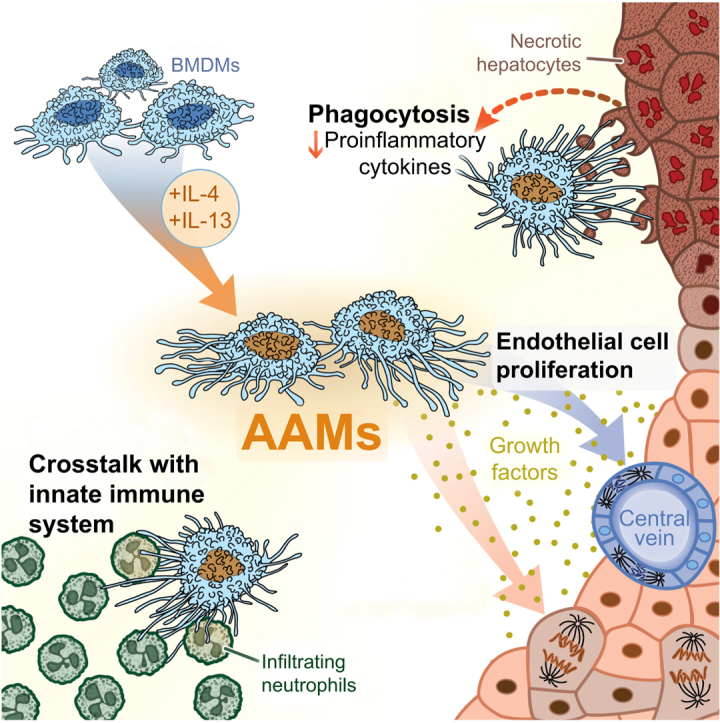

## Introduction

Acetaminophen (paracetamol, APAP) overdose is a common cause of acute liver injury (ALI) in the clinic and is the leading cause of acute liver failure (ALF) in the United States.[1], [2], [3] APAP also serves as a model hepatotoxin for preclinical studies and the molecular mechanisms that underpin APAP hepatotoxicity are relatively well understood. Therapeutic management of APAP-induced ALI (APAP-ALI) is primarily limited to N-acetylcysteine (NAC) therapy, which serves as an effective antidote. However, NAC efficacy is substantially diminished in patients who present late after APAP ingestion (*i.e.* longer than 10 h).^4^ Liver transplantation may be required in patients who subsequently develop ALF. However, due to the shortages of suitable donor tissue, and associated life-long immunosuppression, liver transplantation is not an ideal therapeutic intervention. Therefore, novel therapies to prevent liver injury progressing to ALF are urgently sought.

APAP-ALI is characterised by fulminant hepatocyte necrosis. Without immediate NAC-treatment, substantial liver injury can progress into ALF, which is associated with systemic inflammatory response syndrome (SIRS)—characterised by immune activation and encephalopathy, conferring a high risk of multi-organ failure and death.^5^ Recent work has shown that liver-resident macrophages (Kupffer cells [KCs]), which provide hepatic innate immunity (*e.g.* against gut-derived pathogens), are substantially reduced during APAP-ALI leading to a transient immunological perturbation in the liver.[6], [7], [8] Patients with ALF frequently develop enteric bacterial and fungal infections (typically *Escherichia coli* and *Candida*), which are often associated with fatal outcomes.^9^^,^^10^ The literature is conflicting on the role of macrophages in the pathology of APAP-ALI. Chemical pre-treatment to ablate KCs in rats before APAP-ALI showed protective effects, suggesting macrophages can exacerbate liver injury through release of proinflammatory mediators.[11], [12], [13] However, subsequent studies found that macrophages are absolutely required for appropriate tissue repair and angiogenesis following APAP-ALI.[14], [15], [16] Both KCs and infiltrating macrophages acquire distinct but restorative phenotypes that are required for the timely resolution of APAP-ALI.^6^ Recent work showed that human liver macrophages in patients with ALF also acquire hepatoprotective phenotypes characterised by high phagocytic function and expression of clearance receptors, *e.g.* MERTK.^17^ The clearance of apoptotic and necrotic cells is orchestrated primarily by macrophages, which are fundamentally required to resolve inflammation and injury effectively.^18^^,^^19^ Therefore, we hypothesised that injection of primary macrophages may serve as a cell therapy for APAP-ALI in order to facilitate clearance of necrotic material, reduce local and systemic inflammation, and promote liver regeneration. Primary macrophages can be differentiated from bone marrow precursors *in vitro* to yield a highly enriched population of functional bone marrow-derived macrophages (BMDMs). Injection of BMDMs has previously been shown to ameliorate liver fibrosis in chronic liver injury models.^20^ Clinical-grade autologous human monocyte-derived macrophages (hMDMs) have recently been found safe in cirrhotic patients, with phase II efficacy trials in progress (ISRCTN 10368050).[21], [22], [23]

Herein, we have tested phenotypically distinct BMDM populations in a murine model of APAP-ALI. We report that administration of alternatively activated macrophages (AAMs) reduces necrotic area, reduces several proinflammatory cytokines in tissue and serum, and stimulates hepatocellular proliferation. Importantly from a translational aspect, clinical-grade human AAMs (hAAMs) recapitulated some efficacy readouts in immunocompetent APAP-ALI mice. Our study identifies a potential cell therapy for established APAP-ALI with clinical applicability for a patient group with limited therapeutic options.

## Materials and methods

### BMDM production

BMDMs were prepared as previously, with minor modifications.^20^ Mouse bone marrow (BM) was flushed from femurs and tibias of healthy C57BL/6JOlaHsd male mice (8–10 weeks old, Envigo). BM suspensions were filtered (70 μm) into DMEM:F12 (1:1) cell culture media (Gibco) supplemented with FBS (10%), L-glutamine (2 mM), penicillin/streptomycin (100 U/ml, 100 μg/ml), and murine recombinant CSF1 (40 ng/ml; Peprotech). BM preps were incubated in ultra-low attachment flasks (Corning Inc.) for 7 days (37°C, 5% CO_2_), with additional feeds on days 3 and 5 (20 ng/ml CSF1, in a 50% media change) to produce BMDMs. BMDMs were polarised with recombinant factors overnight to generate classically activated macrophages (CAMs; with lipopolysaccharide [LPS], Sigma Aldrich, 50 ng/ml; and interferon-γ [IFNγ], 20 ng/ml, Peprotech), AAMs (with interleukin [IL]-4 and IL-13; 20 ng/ml each, Peprotech), or deactivated macrophages (DAMs; with IL-10, 10 ng/ml, Peprotech). In some experiments, BMDMs were labelled with CellTrace CFSE (ThermoFisher) *in vitro*, following the manufacturer's instructions.

### hMDM production

For phagocytosis assays, non-GMP hMDMs were differentiated from cryopreserved primary CD14+ monocytes using serum-containing Iscove's Modified Eagle's Medium essentially as described using human recombinant CSF-1 (100 ng/ml, Peprotech).^21^ hCAMs and hAAMs were polarised from hMDMs by overnight stimulation with LPS/hIFNγ (50/20 ng/ml), and hIL-4/IL-13 (20 ng/ml each) respectively. For clinical-grade hMDMs, we utilised a serum-free GMP-compliant process as described.^22^ Peripheral blood mononuclear cells (PBMCs) were centrifuged and collected from healthy volunteer buffy coats using Ficoll-paque 1.077 (GE Healthcare). CD14+ cells were isolated from PBMCs using CliniMACS CD14 MicroBeads (Miltenyi Biotec) on LS separation columns (Miltenyi Biotec). CD14+ cells were cultured (37°C, 5% CO_2_) for 7 days in TexMACS GMP media (Miltenyi Biotec), supplemented with GMP-grade human recombinant CSF-1 (100 ng/ml, R&D Bio-Techne) with an additional feed at day 3 to generate hMDMs. Clinical-grade hAAMs were generated from hMDMs using human recombinant cytokines (R&D Bio-Techne) as above. Successful hMDM differentiation was confirmed using flow cytometry to demonstrate a minimum 5-fold increase in mean fluorescence intensity (MFI) on 25F9 and CD206 compared to initial CD14+ cells.

### APAP-ALI and macrophage administration

Eight-week-old male C57BL6/JOlaHsd mice housed in open top cages were fasted for 14 h. All mice received a single APAP injection (350–500 mg/kg, i.p., Sigma Aldrich) in warm sterile saline (PanReac Applichem). Standard chow and wet mash were returned to mice 20 min post-APAP administration. Macrophages were resuspended in PBS (Sigma) and administered (1–5 × 10^6^ cells, i.v., 100–200 μl) to APAP-ALI mice at 16 h. PBS alone (100–200 μl, i.v.) served as vehicle control for macrophage treatment. After macrophage/vehicle treatment, all mice were transferred to a warming cabinet (28°C). Mice were pulsed with 5-bromo-2′-deoxyuridine (BrdU, 1 mg in sterile saline, i.p., Sigma Aldrich) 1 h before cull to label proliferating cells. Mice were humanely culled and whole blood was collected via cardiac puncture. For immunocompromised mice, the same methodology was used except fasted 12-week old male NOD.Cg-Prkdc^scid^ Il2rg^tm1WjI^/SzJ (NSGs), housed in individually ventilated cages, received 250 mg/kg APAP (i.p.). Substances were administered to NSGs aseptically.

### Statistics

Statistical analysis was performed in Prism 8.2 (GraphPad Software). All data are presented as individual scatter plots to show each experimental unit (*e.g*. individual mice) unless otherwise stated. To test 2 groups, an unpaired 2-way *t* test or Mann-Whitney *U* test was performed on parametric and non-parametric datasets respectively. To test more than 2 parametric groups, a 1-way ANOVA, 2-way ANOVA (with Dunnett's multiple comparison test), or mixed-effects model (with Sidak's multiple comparison's test) was performed. To test more than 2 non-parametric groups, a Kruskal-Wallis test (with Dunn's multiple comparison test) was performed. Shapiro-Wilk test determined normality. Sample size was determined based on power calculation (α = 0.05, desired power = 0.8) or from investigator experience. *p* <0.05 was considered statistically significant.

### Study approval

All animal experiments were undertaken in accordance with criteria outlined in a license granted under the Animals (Scientific Procedures) Act 1986 and approved by the University of Edinburgh Animal Ethics Committee. Use of human material was granted by the South East Scotland Research Ethics Committee 02, and use of buffy coats was covered by Scottish National Blood Transfusion Service (SNBTS). Buffy coats from informed consenting healthy volunteers were obtained in collaboration with SNBTS Blood Donor Centre, Edinburgh, United Kingdom, under SNBTS Sample Governance 16-09.

For further information on flow cytometry, imaging, and other materials and methods, please refer to the CTAT table and supplementary information.

## Results

### AAM-administration reduces hepatic necrosis and stimulates hepatocellular proliferation following APAP-ALI

**Fig. 1:**
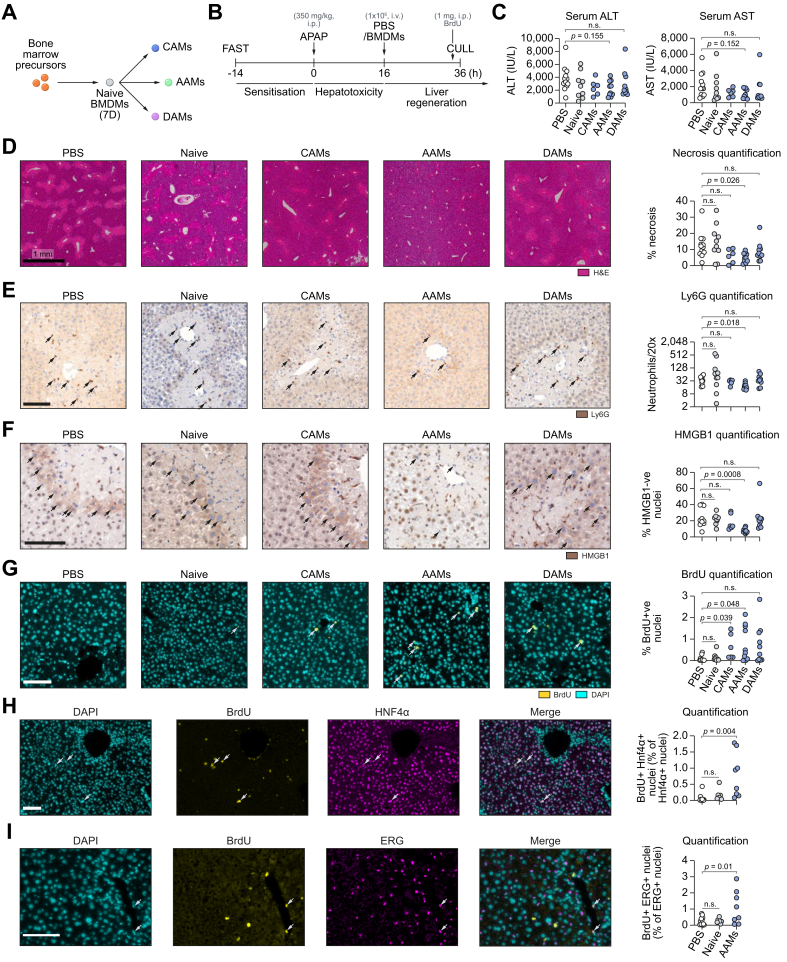
Injection of AAMs reduces necrosis and stimulates liver regeneration following APAP-ALI. (A) Four macrophage populations, derived from mouse BM, were generated for testing: 1. unstimulated BMDMs (naïve), 2. CAMs, 3. AAMs, and 4. DAMs. (B) Study design: injection of macrophages (1 × 10^6^, i.v.) or PBS alone into APAP-ALI mice at 16 h, before cull at 36 h. (C) Serum ALT activity (left panel) and AST activity (right panel) in APAP-ALI mice receiving indicated treatments (D) Representative liver histological stains from APAP-ALI mice receiving indicated treatments, necrosis quantification in right panel (E) Representative Ly6G immunohistochemical stains in liver from APAP-ALI mice with indicated treatments. Black arrows indicate Ly6G-positive cells, quantification in right panel. (F) Representative images of HMGB1 immunohistochemical stains of liver tissue from APAP-ALI mice with indicated treatments. Black arrows indicate HMGB1-negative nuclei, quantification in right panel. (G) Representative immunofluorescence stains of BrdU incorporation (yellow nuclei, indicated by white arrows) in liver with DAPI counterstain (cyan) from APAP-ALI mice with indicated treatments. Quantification in right panel. (H/I) Dual immunofluorescence stains of BrdU (yellow), and either HNF4α (H) or ERG (I) (magenta), with DAPI counterstain (cyan) in AAM-treated liver tissue. White arrows indicate dual-positive cells, quantification in right panel. All data shown are n = 6–12 mice per group (white circles — individual vehicle controls; grey circles — cell-transfer reference group; blue circles — polarised-BMDM-treated mice). Scale bars — 100 μm unless otherwise indicated. *p* values indicated in panels, Kruskal-Wallis test for (C/D/E/F/G/H). n.s., not significant. One-way ANOVA for I. AAMs, alternatively activated macrophages; ALI, acute liver injury; ALT, alanine aminotransferase; APAP, acetaminophen; APAP-ALI, APAP-induced ALI; AST, aspartate aminotransferase; BM, bone marrow; BMDMs, bone marrow-derived macrophages; CAMs, classically activated macrophages; DAMs, deactivated macrophages.

**Fig. 2:**
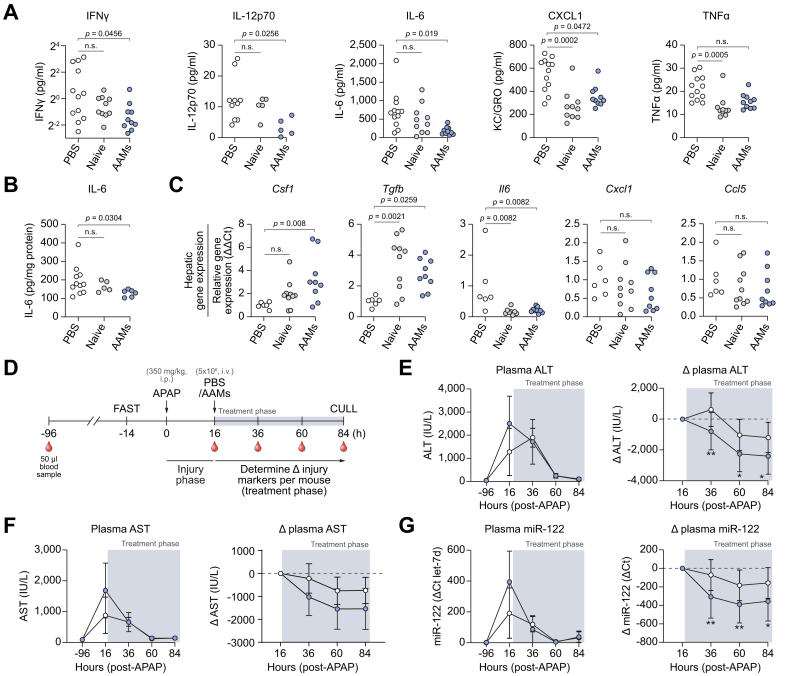
Injection of AAMs reduces several serum proinflammatory cytokines following APAP-ALI. (A) Serum concentrations of proinflammatory cytokines measured in APAP-ALI mice receiving indicated treatments (n = 10–12 per group; some sera had undetectable IL-12p70). (B) IL-6 levels in liver homogenates from APAP-ALI mice receiving indicated treatments (n = 5–11 per group) (C) Relative expression of indicated genes (using 2^−ΔΔCT^ method; standardised to PBS-treated controls, after *GAPDH* normalisation) in liver tissue of APAP-ALI mice receiving indicated treatments (n = 6–10 per group). In A–C, white circles — individual vehicle controls; grey circles — cell-transfer reference group; blue circles — AAM-treated mice. (D) Study design: Plasma biomarkers were measured daily in APAP-ALI mice following PBS/AAM-treatment. (E/F/G) Plasma biomarkers (left panels) and change in plasma biomarkers from point-of-treatment (right panels) for plasma ALT activity (E), AST activity (F), and miR-122 levels (G). Shaded area represents treatment phase. Grouped values represents mean ± SD for AAM-treatment (blue) and PBS-treatment (white). Plasma miR-122 levels are presented as relative quantitation (using 2^-ΔΔCT^ method; standardised to pre-APAP-ALI levels (−96 h), after let-7d normalisation). *p* values provided in panels; n.s., not significant, ∗*p* <0.05, ∗∗*p* <0.01. Kruskal-Wallis tests for A/B/C (*Csf1*/*Ccl5*), one-way ANOVA for C (*Tgfb*, *Il6*, *Cxcl1*), mixed-effects model for (E/F/G). AAMs, alternatively activated macrophages; ALI, acute liver injury; ALT, alanine aminotransferase; APAP, acetaminophen; APAP-ALI, APAP-induced ALI.

First, an appropriate delivery route for injecting BMDMs was identified. We tested whether BMDMs could be injected i.v. to rapidly deliver BMDMs to the liver, since this route is fast, non-invasive, and clinically applicable in the setting of APAP-ALI. *In vivo* and *ex vivo* imaging techniques demonstrated a linear accumulation of BMDMs in the liver and spleen over the first 4 h post-injection (Fig. S1). Next, we tested the efficacy of 4 different macrophage populations (naïve BMDMs, CAMs, AAMs, or DAMs) as a cell-based therapy for APAP-ALI (Fig. 1A; expanded schematic in Fig. S2A). All macrophage populations showed high enrichment for CD11b and F4/80 or CSF1R, and expressed typical markers associated with their activation status (Fig. S2D–G). Each macrophage population (or PBS alone) was administered (1 × 10^6^ cells, 100 μL, i.v.) to mice with APAP-ALI at 16 h when ALI is established (Fig. 1B). Macrophage administration was well tolerated showing no serum chemistry changes in healthy mice (Fig. S3), and no impact on haematological parameters in APAP-ALI mice (Fig. S4). Serum transaminase activity was moderately lower in all BMDM-treated groups compared to PBS-treated controls in APAP-ALI mice, but was not significantly different (Fig. 1C). Serum transaminases have a circulating half-life of several hours,^24^ therefore necrotic area was quantified from H&E stained liver sections for a direct histological measure. AAM-treated mice showed a specific 60% reduction in necrotic area compared to PBS-treated controls in APAP-ALI mice (Fig. 1D). In parallel experiments, AAM treatment reduced necrotic area when injected at 6 h post-APAP at 400 mg/kg (Fig. S5). Neutrophils provide a major source of reactive oxygen species (ROS) that contribute to early injury, although this has been disputed.[25], [26], [27], [28] Macrophages have been implicated in the removal of neutrophils during inflammation.^29^ Neutrophils (detected by Ly6G immunostaining) were 52% lower in necrotic areas specifically in AAM-treated mice compared to PBS-treated controls (Fig. 1E). Next, high-mobility group box 1 (HMGB1), a damage-associated molecular pattern (DAMP), was immunostained in liver sections. Translocation of nuclear HMGB1 into the cytoplasm is an early critical step for its extracellular release.^30^ Peri-necrotic hepatocytes in APAP-ALI showed HMGB1 cytosolic localisation, but the frequency was 66% lower in AAM-treated mice (Fig. 1F). To measure liver regeneration, all mice received BrdU (1 mg, i.p.) 1 h before cull to label proliferating cells at sacrifice. BrdU incorporation in liver tissue was higher in mice treated with CAMs (8.5-fold) and AAMs (8.4-fold) compared to PBS-treated controls (Fig. 1G). These data indicated that AAMs provided the greatest therapeutic response. Therefore, further experiments focused on AAM-treatment, with naïve BMDMs serving as a cell-treatment reference group, and PBS-treatment serving as the vehicle-control group. Dual immunofluorescence (IF) staining revealed proliferating cells after AAM-treatment included both hepatocytes and endothelial cells evidenced by BrdU co-localisation with hepatocyte nuclear factor 4α (HNF4α) and ETS-related gene (ERG) respectively (Fig. 2H, I). Immunostains for isotype antibody controls are provided in Fig. S6. In separate studies, AAMs were tested for efficacy at 500 mg/kg APAP where mortality is expected. Survival experiments are not in compliance with Home Office regulations in the United Kingdom, therefore we established a phenotypic clinical scoring system (see Table S1). Pre-defined thresholds triggered a humane cull in order to prevent mice exceeding severity limits. It was necessary to deliver AAMs at 4 h post-APAP to test efficacy because deaths can occur within 8 h at high APAP doses in mice. We observed no benefit in mice receiving AAMs (1 × 10^6^ cells, i.v.) in this experiment (Fig. S7) suggesting these cells are more efficacious when injected at 16 h.

### AAM-administration reduces several inflammatory cytokines in serum and liver tissue following APAP-ALI

To test the effect on inflammation after AAM-treatment, a panel of cytokines were quantified in serum and liver homogenates obtained from APAP-ALI mice. Consistently, AAM treatment specifically reduced several serum proinflammatory cytokines including IFNγ (82%), IL-12p70 (73%), and IL-6 (75%) *vs.* PBS-treated controls (Fig. 2A). Treatment with both AAMs and naïve BMDMs reduced serum CXCL1 levels. Serum tumour necrosis factor-α was 39% lower after BMDM treatment suggesting that naïve BMDMs may exert subtle effects on circulating cytokines. Importantly, hepatic IL-6 levels were 34% lower specifically in AAM-treated liver homogenates compared to PBS-treated controls (Fig. 2B). Further, we measured the expression of a panel of genes associated with inflammation in whole liver tissue. We observed 3.3-fold higher expression of *Csf1* specifically in AAM-treated mice *vs.* PBS-treated controls, whilst higher *Tgfb* and lower *Il6* expression levels were observed in both AAM- and naïve BMDM-treated mice (Fig. 2C). Expression of other inflammatory-associated genes such as *Cxcl1* and *Ccl5* were lower on average in AAM-treated liver but did not reach statistical significance. To better understand the trajectory of recovery following AAM-treatment, a longitudinal experiment was performed in APAP-ALI mice by performing serial blood microsampling up to 84 h (study design: Fig. 2D). Plasma levels of alanine aminotransferase (ALT), aspartate aminotransferase (AST), and the hepatocyte-specific microRNA, miR-122, showed significantly bigger reductions after AAM-treatment compared to PBS-treated controls suggesting improved recovery (Fig. 2E–G).

### Murine AAMs are primarily Ly6C^lo^ and highly phagocytic in APAP-ALI liver tissue post-injection

**Fig. 3:**
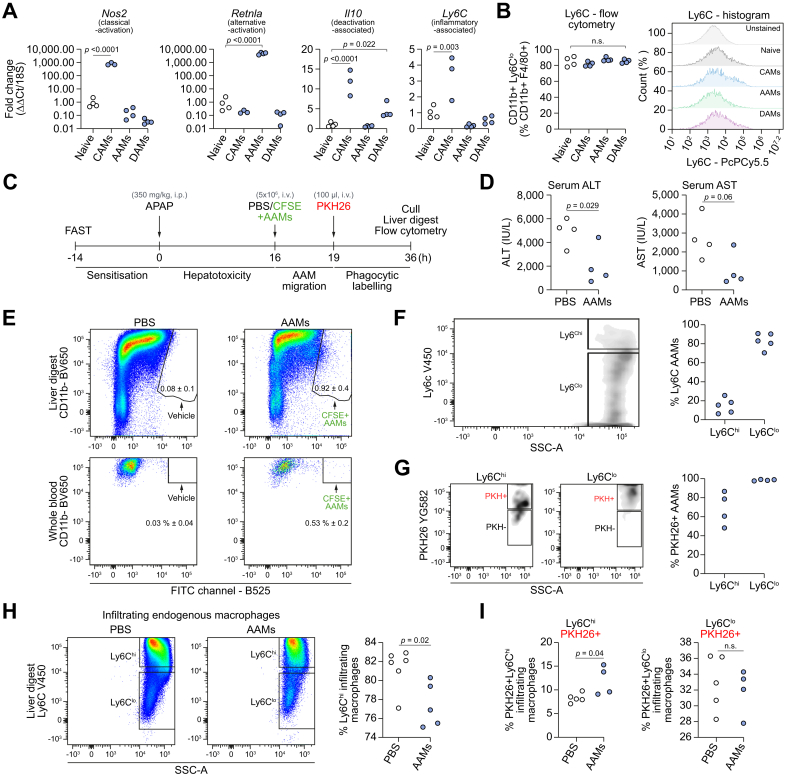
Injected AAMs are primarily Ly6C^lo^ and highly phagocytic *in situ*. (A) Relative gene expression in BMDM populations (determined by 2^−ΔΔCT^ method; standardised to naïve BMDMs, after *18S* normalisation): *Nos2* — CAM-associated gene, *Retnla* — AAM-associated gene, *Il10* — DAM-associated gene, *Ly6C* — proinflammatory-associated gene (n = 3/4 biological replicates per group). (B) Ly6C status in BMDM populations (flow cytometry quantification, left panel; cytometry histograms, right panel) (C) Study design: CFSE-labelled AAMs injected (5 × 10^6^, i.v.) at 16 h in APAP-ALI mice, 3 h before PKH26 (fluorescent phagocytic tracer). Cull at 36 h. (D) Serum ALT activity (left) and AST (right) in APAP-ALI mice treated with PBS or AAMs (n = 4 per group). (E) Gating shows CFSE+ AAMs in liver digests (top panels) and whole blood (bottom panels) in AAM-treated APAP-ALI mice. (F). Representative flow plot of Ly6C status in retrieved CFSE+ AAMs (gating: left panel; quantification: right panel, circles represent digests from individual mice). (G) Representative flow plots showing PKH uptake in Ly6C^hi^ (left panel) and Ly6C^lo^ (middle panel) AAMs, quantification in right panel. (H) Representative flow plots show Ly6C status in infiltrating endogenous macrophages in liver digests from APAP-ALI mice treated with PBS (left panel) or AAMs (middle panel), quantification in right panel. (I) Quantification of PKH uptake in Ly6C^hi^ (left panel) and Ly6C^lo^ (right panel) infiltrating endogenous macrophages. *p* values provided in panels, n.s. not significant. One-way ANOVA (A), unpaired *t* test (D, I), or Mann-Whitney *U* test (H) were performed. AAMs, alternatively activated macrophages; ALI, acute liver injury; ALT, alanine aminotransferase; APAP, acetaminophen; APAP-ALI, APAP-induced ALI; AST, aspartate aminotransferase; BM, bone marrow; BMDMs, bone marrow-derived macrophages; CAMs, classically activated macrophages; DAMs, deactivated macrophages.

A series of experiments was performed to assess the phenotype and function of AAMs *in vitro* and *in vivo*. Gene expression analysis confirmed polarisation occurred *in vitro* for all BMDM populations, *e.g. Nos2* was 775-fold higher in CAMs, *Retnla* was 4,850-fold higher in AAMs, whilst *Il10* and *Ly6C* expression was higher in DAMs and CAMs, respectively (Fig. 3A; further data in Fig. S2B). Flow cytometry revealed that all BMDM subsets were approximately 80% Ly6C^lo^ *in vitro* (Fig. 3B). To investigate AAM phagocytosis *in vivo*, CFSE-labelled AAMs were administered (5 × 10^6^, i.v.) at 16 h, 3 h before administration of PKH26PCL (PKH, i.v.), a fluorescent probe that specifically labels cells performing phagocytosis (Fig. 3C). A higher number of AAMs were injected in this experiment to improve assay sensitivity. Serum ALT activity was significantly lower in AAM-treated mice compared to PBS-treated controls (Fig. 3D). A flow cytometry gating strategy was used to analyse myeloid cells in liver digests (Fig. S8). CFSE+ AAMs represented 0.9% and 0.5% of the CD11b+ population in liver digests and whole blood respectively in APAP-ALI mice (Fig. 3E). Low expression of Ly6C, a cell surface myeloid marker, has been associated with a restorative macrophage phenotype during liver disease.^31^ Injected AAMs remained approximately 80% Ly6C^lo^ at 36 h in APAP-ALI mice (Fig. 3F). Furthermore, AAMs were highly phagocytic showing high PKH uptake in Ly6C^hi^ (69% positive) and Ly6C^lo^ (99% positive) AAMs (Fig. 3G). We observed a 5% reduction in infiltrating endogenous Ly6C^hi^ macrophages in AAM-treated mice (Fig. 3H). Furthermore, PKH uptake in Ly6C^hi^ infiltrating endogenous macrophages increased from 8.3% in PBS-treated controls to 12% in AAM-treated mice, whilst PKH uptake was equivalent in Ly6C^lo^ infiltrating macrophages (Fig. 3I) suggesting crosstalk exists between AAMs and the host innate immune response. Finally, to test if AAMs alter their phenotype post-injection, we performed a low-density PCR array on FACS-sorted AAMs from healthy and APAP-ALI liver digests. We found only 4 genes (*Il10*, 15.3-fold; *C4b,* 8.8-fold, *Tlr4* 6.1-fold; and *B2m,* 6.2-fold) from 84 tested genes were downregulated in AAMs after injection into APAP-ALI mice compared to injection into healthy mice (Fig. S9A–D and Table S2). These data suggest that injected AAMs are highly phagocytic *in situ*, and largely retain their phenotype post-injection.

### Murine AAMs are highly phagocytic *in vitro*

**Fig. 4:**
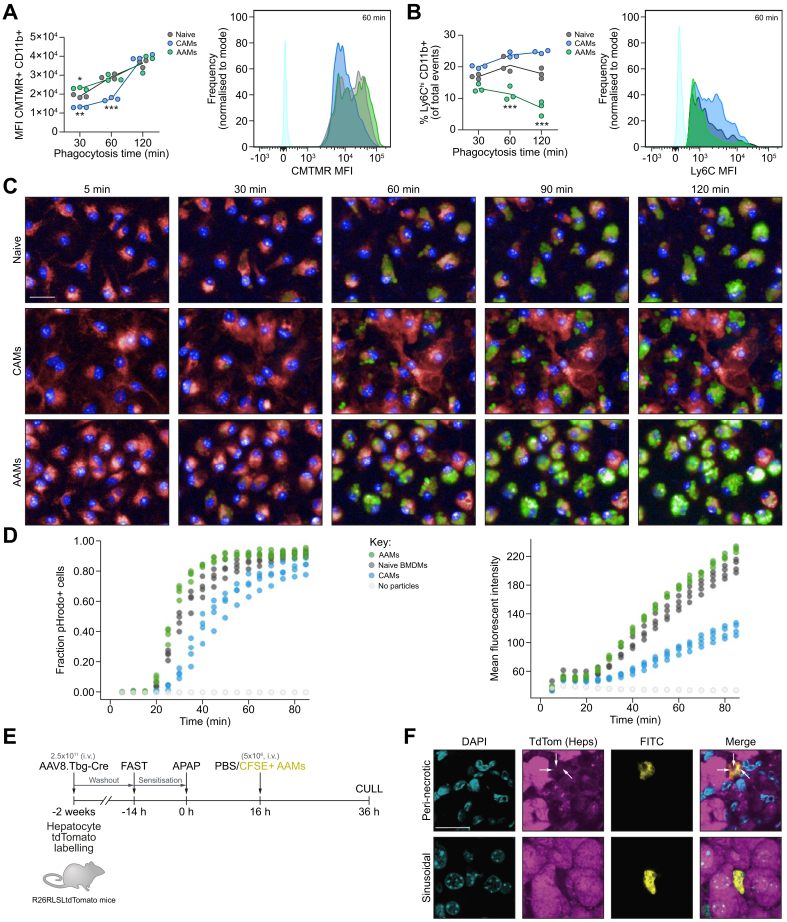
Murine AAMs are highly phagocytic. (A) Phagocytosis quantification in naïve BMDMs (dark grey), AAMs (green), or CAMs (blue) during incubation with CMTMR-labelled apoptotic thymocytes. MFI of CMTMR-positive macrophage populations (left), and representative histograms (right). (B) Ly6C status in BMDM populations during incubation with CMTMR-labelled apoptotic thymocytes at indicated times (left panel); representative histograms (right panel). Coloured circles represent individual preparations connected by lines (mean value, n = 3). (C) Representative images of real-time phagocytosis at indicated times. Naïve BMDMs (top row), CAMs (middle row) and AAMs (bottom row) are shown (Deep Red CellMask, red; NucBlue, blue). Phagocytosis indicated by intracellular fluorescence (green). (D) Phagocytosis quantification: pHrodo-positive cell fraction (left) and total cell MFI (right). (E) Study design: Hepatocytes were Tdtomato-labelled by delivery of hepatotropic AAV8 virus delivering Cre-recombinase to R26RLSLtdTomato mice. Tdtomato-positive APAP-ALI mice received CFSE-labelled AAMs (5 × 10^6^, i.v.) at 16 h, before cull (36 h). (F) Panels show representative confocal immunofluorescence of liver tissue (max intensity projection from 7 slices; 2.4 μm) in each channel: DAPI (cyan), TdTom (TdTomato+ hepatocytes, magenta), FITC (CFSE+ AAMs, yellow), and merged images. Faint punctate TdTomato+ debris were visible inside vesicles in peri-necrotic macrophages (top row, white arrow heads). ∗*p* <0.05, ∗∗*p* <0.01, ∗∗∗*p* <0.001, scale bars 20 μm. Two-way ANOVA (A/B). AAMs, alternatively activated macrophages; AAV, adeno-associated virus; ALI, acute liver injury; APAP, acetaminophen; APAP-ALI, APAP-induced ALI; AST, aspartate aminotransferase; BM, bone marrow; BMDMs, bone marrow-derived macrophages; CAMs, classically activated macrophages; DAMs, deactivated macrophages; MFI, mean fluorescence intensity.

To further understand the phenotype and function of AAMs, we performed a series of *in vitro* phagocytosis experiments. The propensity of BMDM populations to phagocytose apoptotic material was assessed by flow cytometry after incubating BMDMs with (5-(and-6)-(((4-chloromethyl)benzoyl)amino)tetramethylrhodamine)- (CMTMR-) labelled apoptotic thymocytes using a gating strategy (Fig. S10). The MFI was higher in AAMs at 30 min *vs.* other groups (21% increase *vs.* naïve, 75% increase *vs.* CAMs) and at 60 mins *vs.* CAMs (64% increase; Fig. 4A). Furthermore, AAMs had a significantly lower percentage of Ly6C^hi^ cells at 30 min (22% lower *vs.* naïve, 34% *vs.* CAMs), 60 min (44% lower *vs.* naïve, 52% *vs.* CAMs), and 120 min (61% lower *vs.* naïve, 72% *vs.* CAMs) suggesting AAMs possess a sustained phenotype throughout phagocytosis (Fig. 4B). In parallel, real-time live imaging assays showed AAMs could phagocytose pHrodo-bioparticles faster and to a greater extent than other BMDM populations (Fig. 4C, D, and Video S1). Similarly, hAAMs produced from cryopreserved human CD14+ cells also showed similar enhanced phagocytic performance *in vitro* (Fig. S11 and Video S2). To test if AAMs could phagocytose necrotic hepatocytes *in vivo*, CFSE+ AAMs (5 × 10^6^, i.v.) were injected into APAP-treated R26RLSL tdTomato mice 2 weeks after viral-Cre delivery to induce tdTomato in hepatocytes (Fig. 4E). Injected AAMs localised throughout the liver parenchyma, with many surrounding necrotic areas (Fig. S12A). Labelled mice developed APAP-ALI characterised by a loss of tdTomato hepatocytes around the central vein and raised serum ALT activity (Fig. S12A, B). Confocal microscopy revealed that peri-necrotic AAMs contained multiple intracellular vesicles containing faint fluorescent punctate tdTomato-positive material consistent with surrounding hepatocellular debris (Fig. 4F). In contrast, no tdTomato material was visible in sinusoidal AAMs located distal to necrotic areas in the same tissue.

### Administration of human AAMs reduces necrosis and stimulates hepatocyte proliferation following APAP-ALI in immunocompetent mice

**Fig. 5:**
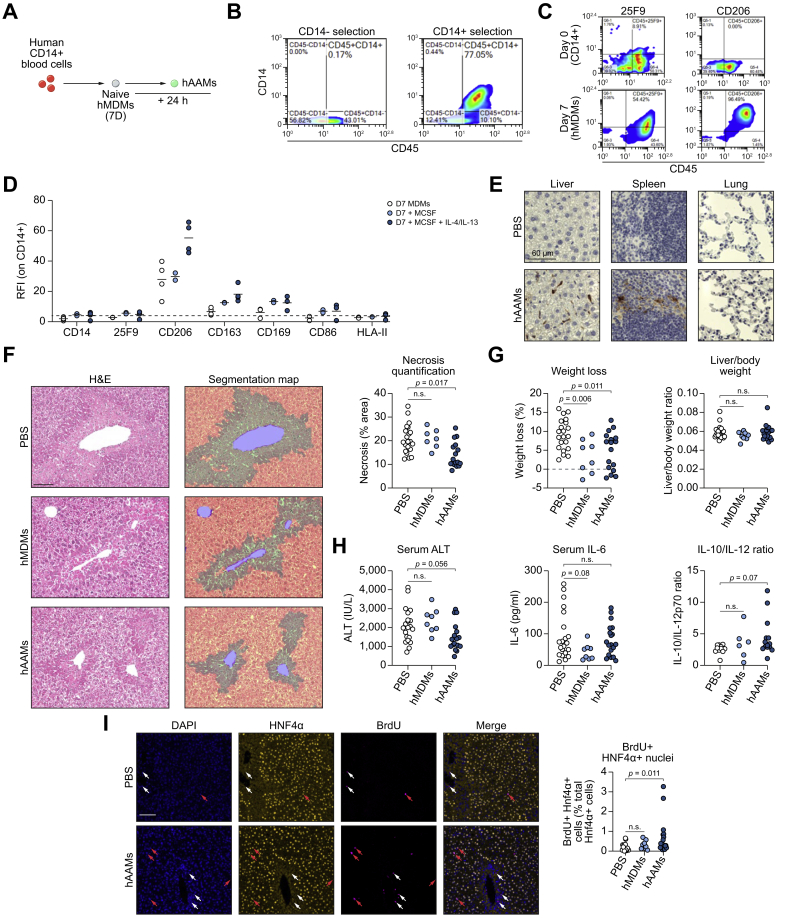
Injection of hAAMs reduces necrosis in APAP-ALI immunocompetent mice. (A) hMDMs were differentiated from CD14+ cells isolated from healthy volunteer buffy coats, before incubation with hCSF-1 for 7 days. hAAMs were generated by stimulating hMDMs overnight with hCSF1, hIL-4, and hIL-13. (B) Representative flow cytometry plots demonstrating CD14-enrichment using CliniMACS® beads (C) Representative flow cytometry plots showing macrophage maturity markers (25F9, left panels; CD206, right panels) in CD14+ cells (top) and hMDMs (bottom) (D) Flow cytometry quantification of hMDMs (white), hMDMs stimulated with CSF-1 alone (light blue), and fully-stimulated hAAMs (dark blue), spots represent individual donors. (E) Panels show representative anti-FITC immunohistochemical stains in liver (left), spleen (centre), and lung (right) from APAP-ALI mice treated with PBS (top row) or hAAMs (bottom row). (F) Representative histological staining (left) and necrosis segmentation map (centre) in APAP-ALI mice with indicated treatments. Necrosis quantification in right panel (n ≥7 mice per group). (G) Percentage weight loss (left panel) and liver/body weight ratio (right panel) of APAP-ALI mice with indicated treatments (n ≥8 per group) (H). Serum injury/inflammatory markers in APAP-ALI mice receiving indicated treatments (n ≥6 per group; serum ALT activity, left panel; serum IL-6, centre panel; serum IL-10/-12p70 ratio, right panel, some sera has undetectable IL-12p70 levels). (I) Representative dual immunofluorescence images of HNF4α (yellow) and BrdU (magenta) against DAPI (counterstain, blue) in liver tissue from APAP-ALI mice treated with PBS (top row) or hAAMs (bottom row). White arrowheads indicate BrdU-positive cells, red arrowheads indicate dual-positive BrdU-positive HNF4α-positive cells (quantification right panel; n≥ 8). Scale bars 100 μm, unless otherwise indicated. *p* values indicated in panels, n.s. not significant. One-way ANOVA test in F, G (weight loss %), H (ALT), or Kruskal-Wallis test in G (liver/body ratio), H (IL-6, IL-10/-12 ratio), and I. ALI, acute liver injury; APAP, acetaminophen; APAP-ALI, APAP-induced ALI; hAAMs, alternatively activated macrophages; hMDMs, human monocyte-derived macrophages.

A fully defined serum-free protocol to generate clinical-grade hMDMs has recently been described.^22^ Here, we modified this protocol to generate clinical-grade hAAMs for evaluation in the APAP-ALI model (Fig. 5A; expanded schematic Fig. S13A). Isolation of CD14+ cells using CliniMACS microbeads from human PBMCs provided significant CD14+ CD45+ enrichment (Fig. 5B). CD14+ cells were cultured with GMP-grade recombinant hCSF-1 to generate hMDMs. Flow cytometry revealed hMDMs upregulated human macrophage maturity markers 25F9 and CD206 after 7 days (Fig. 5C). hMDMs were stimulated with hIL-4/-13 *in vitro* for 24 h to generate hAAMs, exhibiting higher CD206, CD163 and CD169 surface levels (Fig. 5D). hAAMs had higher *MRC1*, *DC-SIGN*, *SCARB1* gene expression, and lower *NOS2* compared to volunteer-matched hMDMs (Fig. S13B). Firstly, hAAMs were tested in an immunocompromised APAP-ALI model (1 × 10^6^, i.v., NSG mice, 250 mg/kg). NSG mice could only tolerate mild centrilobular necrosis, therefore a lower APAP dose was used (250 mg/kg). However, hAAMs were not efficacious in the NSG model; there was no change in necrosis or liver injury markers (Fig. S14). Therefore, we re-tested hAAMs (5 × 10^6^, i.v.) in an immunocompetent strain (C57BL/6J) with APAP-ALI. Firstly, healthy C57BL/6J mice tolerated hMDM injection well, showing no changes in body weight, blood circulating monocytes or blood neutrophils at 24 h or 7 days post-injection with no phenotypic evidence of severe acute rejection (Fig. S15). Human cells were expected to be rapidly cleared in immunocompetent mice. Nevertheless, some FITC-positive cells were detected in the liver and spleen of APAP-ALI mice treated with CFSE-labelled AAMs, suggesting transient hepatic localisation is possible (Fig. 5E). Histological evaluation showed 32% reduced necrosis specifically in hAAM-treated mice (Fig. 5F). Both hMDM and hAAM treatment led to reduced weight loss, which was not related to any changes in liver mass indicated by consistent liver/body weight ratios (Fig. 5G). Like murine AAM treatment, serum ALT activity showed a strong reduced trend in hAAM-treated mice compared to PBS-treated controls, but did not reach statistical significance (serum ALT, *p* = 0.056; Fig. 5H). We observed no changes in serum cytokines, including murine IL-6, in hAAM-treated mice *vs.* PBS-controls, although murine IL-10/IL-12 ratio displayed a higher trend in hAAM-treated mice (*p* = 0.07, Fig. 5H). Importantly, an increase in BrdU incorporation was observed in HNF4α-positive nuclei specifically in hAAM-treated liver tissues (Fig. 5I) suggesting improved hepatocyte proliferation. These data suggest that hAAMs can recapitulate some efficacy readouts of murine AAMs in experimental APAP-ALI.

## Discussion

NAC is the primary treatment option for APAP overdoses and acts through boosting the antioxidant capacity of the liver to prevent liver injury occurring. NAC efficacy is diminished in patients who present to hospital late (*i.e.* 10–12 h after APAP ingestion) when liver injury may already be established. Therefore, novel therapeutic strategies are required to treat late-presenting APAP-ALI patients with established liver injury. Here, we demonstrate a novel cell-based immunotherapeutic approach to promote necrosis resolution, reduce systemic inflammation, and expedite liver repair in experimental APAP-ALI. Recent work has shown that KCs, highly phagocytic liver-resident macrophages, are depleted during APAP-ALI impairing hepatic innate immunity.^6^ Large numbers of monocytes rapidly infiltrate the liver thereafter and differentiate into monocyte-derived macrophages *in situ*, but they are inflammatory (*e.g*. Ly6C^hi^) and poorly phagocytic, at least initially.^6^^,^^32^^,^^33^ Therefore, we hypothesised that timely intervention with polarised macrophages may hold therapeutic value as a cell-based therapy for ALI to facilitate necrosis resolution and promote liver regeneration. Macrophages have previously been evaluated as an experimental cell-based therapy for other diseases,^34^^,^^35^ including liver fibrosis,^20^ but have not yet been tested in the setting of ALI.

Hepatocyte necrosis during APAP-ALI is the priming event to activate the innate immune system, through release of proinflammatory cytokines and DAMPs, *e.g.* HMGB1.^36^ We observed that AAM-treatment specifically reduced necrotic area in APAP-ALI mice within 24 h. AAMs also led to reductions in infiltrating neutrophils, cytosolic HMGB1 translocation (in peri-necrotic hepatocytes), and attenuated several serum proinflammatory cytokines suggesting AAMs exert an anti-inflammatory effect. This has potential clinical importance because systemic inflammation (*via* uncontrolled activation of the innate immune system) is a feature of SIRS—a key determinant of clinical outcome in APAP-ALF.^5^ Furthermore, AAM-treatment also led to improved hepatic proliferation, particularly in hepatocytes and endothelium. Hepatic proliferation and revascularisation are recognised as important features of liver regeneration following injury.[37], [38], [39]

AAM efficacy was associated with a highly phagocytic phenotype, therefore we posited that AAM delivery may augment clearance of necrotic material in APAP-ALI since host KCs are depleted. AAMs accounted for 0.9% of the liver myeloid population 20 h post-transfer in APAP-ALI mice. AAMs were highly phagocytic both in culture and in liver tissue (80% Ly6C^lo^, >99% PKH uptake). Furthermore, phagocytosis was poor (8.2% PKH uptake) in endogenous Ly6C^hi^ infiltrating macrophages in APAP-ALI mice but improved with AAM-treatment (12% PKH uptake). This suggests that AAM treatment can modulate the dynamics of the host immune response amplifying the therapeutic effect. Consistent with this, AAMs were ineffective when delivered at 4 h post-APAP in the high-dose APAP (500 mg/kg) experiment, *i.e.* before CCR2+ monocytes infiltrate the liver.^32^ Indeed, hAAMs were ineffective in immunocompromised mice, suggesting an immunocompetent system is required to achieve efficacy. In immunocompetent mice, hAAMs improved necrosis resolution and hepatocyte proliferation (albeit requiring a higher number of transferred cells). However, hAAMs had no effect on serum proinflammatory cytokine levels, possibly due to rapid clearance of human cells by host natural killer cells, or the lack of host response from any hAAM-derived factors due to species differences in immune signalling pathways. AAM efficacy is therefore likely to be attributed to both the phagocytosis of necrotic tissue and paracrine effects upon the host immune system.

Mouse and human AAMs took 8 days to generate using current protocols—a timeframe that is clinically incompatible with the emergency settings of an APAP overdose. A potential clinical therapeutic product would therefore likely be allogenic, immunocompatible, scalable, and permit cryopreservation so that cells could be rapidly administered in an emergency scenario. In summary, AAM therapy for ALI reduces necrosis and inflammation and increases liver regeneration and is a potential novel therapy for late-presenting APAP-ALI.

### Abbreviations

AAMs, alternatively activated macrophages; AAV, adeno-associated virus; ALF, acute liver failure; ALI, acute liver injury; ALT, alanine aminotransferase; APAP, acetaminophen; APAP-ALI, APAP-induced ALI; AST, aspartate aminotransferase; BM, bone marrow; BMDMs, bone marrow-derived macrophages; CAMs, classically activated macrophages; DAMs, deactivated macrophages; DAMP, damage-associated molecular pattern; hAAMs, human AAMs; hMDMs, human monocyte-derived macrophages; IFN, interferon; IL, interleukin; LPS, lipopolysaccharide; MFI, mean fluorescence intensity; PBMCs, peripheral blood mononuclear cells; ROS, reactive oxygen species.

## Financial support

P.S.L. was supported by grant funding from the UK Regenerative Medicine Platform (UKRMP) Safety and Efficacy Hub (grant ref MR/K026739/1), and Stem Cell Niche Hub. Human macrophage studies were supported by an MRC Confidence in Concept award (MRC/CIC5/06), with research collaboration funding from Syncona Ltd. (funder reference: MED2589). L.C. was supported by 10.13039/501100000265MRC grant (MR/J010766/1). T.J.K. was supported by Wellcome Trust Intermediate Clinical Fellowship (095898/Z/11/Z). J.C. was supported by 10.13039/100004440Wellcome Trust grant (10896/Z/15/Z). N.A. was supported by 10.13039/100004440Wellcome Trust grant (203925/Z/16/A). Funding sources had no involvement in study design, data collection, data analysis and interpretation, writing of the report, nor decision to publish.

## Authors' contributions

S.J.F. and P.S.L. conceived the study concept and experimental design. P.S.L., L.C., J.C., A.M., A.T., V.G., R.A., and T-Y.M. performed experiments. P.S.L., L.C., N.A., J.C., and B.D, collected data and performed statistical analysis. T.K., E.O., and M.V. provided analytical and imaging support. A.R., L.F., and K.P. provided intellectual input and supervision. P.S.L., L.C., and S.J.F. drafted the manuscript. All authors reviewed and edited the manuscript.

## Conflict of interest

P.S.L., L.F., and S.J.F. have patents pending, entitled ‘Macrophage-based therapy’ in national territories of USA, Europe, Japan, China and Australia. These patents have been derived from PCT/GB2017/052769 filed 18/09/2017 and claim priority from UK application 1615923.8 filed 19/09/2016. Both of the original patents have now been abandoned because the original UK patent and PCT patent are no longer live and have now been replaced by the national patents.

Please refer to the accompanying ICMJE disclosure forms for further details.

## Supplementary data

CTAT table.pdf
