# Supplementary material for: Alternatively activated macrophages promote resolution of necrosis following acute liver injury
Source: J Hepatol. 2020 Aug;73(2):349–60. doi: 10.1016/j.jhep.2020.02.031 (PMC7378576; doi:10.1016/j.jhep.2020.02.031)
Supplement: CTAT table.pdf [file mmc1.pdf]

JOURNAL
OF HEPATOLOGY

Journal of Hepatology

CTAT methods

Tables for a "Complete, Transparent, Accurate and Timely account" (CTAT) are now mandatory for all revised submissions. The aim is to enhance the reproducibility of methods.

- Only include the parts relevant to your study
- Refer to the CTAT in the main text as 'Supplementary CTAT Table'
- Do not add subheadings
- Add as many rows as needed to include all information
- Only include one item per row

If the CTAT form is not relevant to your study, please outline the reasons why:

1.1 Antibodies

Name	Citation	Supplier	Cat no.	Clone no.
**IHC/IF assays**				
Anti-FITC		ThermoFisher	71-1900	Polyclonal
Anti-BrdU		Abcam	ab6326	BU1/75 (ICR1)
Anti-HMGB1		Abcam	ab18256	Polyclonal
Anti-HNF4α		Perseus Proteomics	PP-H1415-00	H1415
Anti-Ly6G		BioLegend	127602	1A8
Anti-ERG		Abcam	ab92513	EPR3864
Anti-mCherry (recognises tdTomato)		SICGEN	AB0081-500	Polyclonal
Anti-CD68		Abcam	ab213363	EPR20545
**Flow cytometry conjugated antibodies (anti-mouse, phagocytosis studies)**				
CD11b (BV650)		eBioscience	64-0112-82	M1/70
CD11b FITC		eBioscience	11-0112-41	M1/70
Ly-6C V450		eBioscience	48-5932-82	HK1.4
Ly-6C PerCP/Cy5.5		eBioscience	45-5932-82	HK1.4
CD45.2 AF700		eBioscience	56-0454-82	104
F4/80 APC		Invitrogen	MF48005	BM8
Ly-6G PE-Cy7		BioLegend	127618	1A8
CD3 PE-Cy7		BioLegend	100220	17A2
NK1.1 PE-Cy7		BioLegend	108714	PK136
CD19 PE-Cy7		BioLegend	115520	6D5
CD31 PE-Cy7		eBioscience	25-0311-82	390
**Flow cytometry conjugated antibodies (anti-mouse, phenotyping)**				
F4/80 APC		BioLegend	123115	BM8
CD11b BV650		BioLegend	101239	M1/70
Ly6G BV421		BioLegend	127627	1A8
Ly6C PerCP/Cy5.5		BioLegend	128011	HK1.4
CD115 PE		BioLegend	135505	AFS98
CD71 BV421		BioLegend	113813	RI7217

CD86 BV421		BioLegend	105031	GL-1
CD83 FITC		BioLegend	121505	Michel-19
I-Ab PerCP/Cy5.5		BioLegend	116405	AF6-120.1
CD206 FITC		BioLegend	141703	C068C2
CD11c		BioLegend	371503	S-HCL-3
**Flow cytometry conjugated antibodies (human)**				
CD45 VioBlue		Miltenyi Biotec	130-113-122	5B1
CD14 PE		Miltenyi Biotec	130-091-242 (130-113-147)	TÜK4
CD14 VioBlue		Miltenyi Biotec	130-113-152	TÜK4
CD206 FITC		Miltenyi Biotec	130-095-131	DCN228
25F9 APC (eFluor660)		eBioscience	50-0115-42	25F9
CCR2 PE		BioLegend	357206	K036C2
CD163 FITC		Miltenyi Biotec	130-097-626	GHI/61.1
CD169 APC		BioLegend	346008	7-239
CD86 PE		BioLegend	374206	BU63
HLA FITC		BioLegend	361705	Tu39

1.2 Cell lines

Name	Citation	Supplier	Cat no.	Passage no.	Authentication test method

1.3 Organisms

Name	Citation	Supplier	Strain	Sex	Age	Overall n number
Mice (wild-type)		Harlan	C57CBL/6J OlaHsd	Male	8-10 weeks old	N=3-11 per group (experiment-dependent)
Mice (Immunocompromised)		Charles River	NSG - NOD.Cg-PrkdcSCID Il2rgtm1Wjl/SzJ	Male	12 weeks old	N=9/10 per group

1.4 Sequence based reagents

Name	Sequence	Supplier

1.5 Biological samples

Description	Source	Identifier

1.6 Deposited data

Name of repository	Identifier	Link

1.7 Software

Software name	Manufacturer	Version

1.8 Other (e.g. drugs, proteins, vectors etc.)

Acetaminophen (BioXtra)	Sigma Aldrich	A7085

1.9 Please provide the details of the corresponding methods author for the manuscript:

Philip Starkey Lewis, PhD
MRC Centre for Regenerative Medicine
University of Edinburgh,
5 Little France Drive,
Edinburgh,
EH16 4UU

Email: pstarkey@staffmail.ed.ac.uk

2.0 Please confirm for randomised controlled trials all versions of the clinical protocol are included in the submission. These will be published online as supplementary information.

