# Supplementary material for: Alternatively activated macrophages promote resolution of necrosis following acute liver injury
Source: J Hepatol. 2020 Aug;73(2):349–60. doi: 10.1016/j.jhep.2020.02.031 (PMC7378576; doi:10.1016/j.jhep.2020.02.031)
Supplement: Supplementary information.pdf [file mmc3.pdf]

Alternatively activated macrophages promote resolution of necrosis following acute liver injury

Philip Starkey Lewis, Lara Campana, Niya Aleksieva, Jennifer Ann Cartwright, Alison Mackinnon, Eoghan O'Duibhir, Timothy Kendall, Matthieu Vermeren, Adrian Thomson, Victoria Gadd, Benjamin Dwyer, Rhona Aird, Tak-Yung Man, Adriano Giorgio Rossi, Lesley Forrester, B. Kevin Park & Stuart John Forbes

Table of contents

Fig. S1……………………………………………………………………………......3

Fig. S2……………………………………………………………………………..5

Fig. S3……………………………………………………………………………..7

Fig. S4…………………………………………………………………………..…8

Fig. S5……………………………………………………………………………..9

Fig. S6……………………………………………………………………………11

Fig. S7……………………………………………………………………………13

Fig. S8……………………………………………………………………………14

Fig. S9…………………………………………………………………………...16

Fig. S10…………………………………………………………………………18

Fig. S11…………………………………………………………………………19

Fig. S12…………………………………………………………………………20

Fig. S13…………………………………………………………………………22

Fig. S14……………………………………………………………………….....23

Fig. S15……………………………………………………………………….....24

Table S1………………………………………………………………………....25

Table S2……………………………………………………………………………………26

Table S3……………………………………………………………………………………27

Table S4……………………………………………………………………………………28

Supplementary methods…………………………………………………………………30

Supplementary references……………………………………………………………… 38

Fig. S1

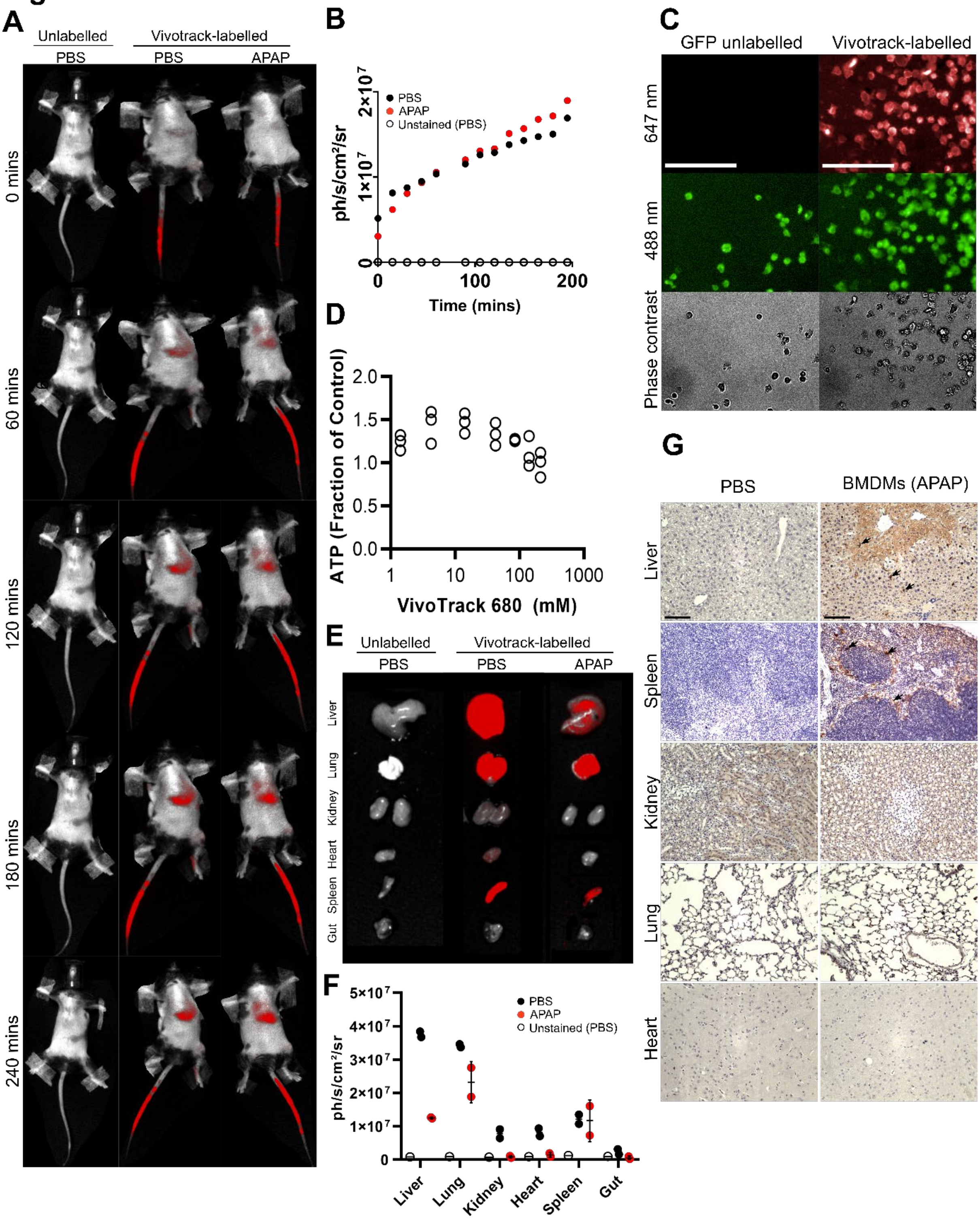

A
Unlabelled
Vivotrack-labelled
PBS
PBS
APAP
0 mins
60 mins
120 mins
180 mins
240 mins
B
PBS
APAP
Unstained (PBS)
ph/s/cm²/sr
2x10^7
1x10^7
0
0
100
200
Time (mins)
C
GFP unlabelled
Vivotrack-labelled
647 nm
488 nm
Phase contrast
D
ATP (Fraction of Control)
2.0
1.5
1.0
0.5
0.0
1
10
100
1000
VivoTrack 680 (mM)
E
Unlabelled
Vivotrack-labelled
PBS
PBS
APAP
Liver
Lung
Kidney
Heart
Spleen
Gut
F
PBS
APAP
Unstained (PBS)
ph/s/cm²/sr
5x10^7
4x10^7
3x10^7
2x10^7
1x10^7
0
Liver
Lung
Kidney
Heart
Spleen
Gut
G
PBS
BMDMs (APAP)
Liver
Spleen
Kidney
Lung
Heart

Fig. S1. BMDMs rapidly engraft in liver and spleen after i.v. injection. (A) Live infrared fluorescent imaging of anaesthetised mice shows Vivotrack-labelled BMDMs ($5x10^6$, i.v.) accumulating in the upper abdomen (red signal) over 240 minutes in healthy and APAP-ALI mice. (B) Quantification of abdominal fluorescence in individual mice at 15 minute intervals. Open circles indicate a representative mouse receiving unstained BMDMs, closed circles indicate a representative mouse receiving Vivotrack-stained BMDMs (black circles, healthy mouse; red circles, APAP-ALI mouse). (C) IF microscopy shows uniform labelling of Vivotrack-labelled GFP+ BMDMs (right-hand panels) versus unlabelled GFP+ BMDMs (left-hand panels) at 647 nm (top row). GFP+ BMDMs visualised at 488 nm (middle panels) and brightfield (bottom panels) (D) Relative ATP levels in BMDMs after Vivotrack labelling. Open circles represent three individual labelling preparations. (E) Ex vivo fluorescent imaging of individual organs 4 hours after BMDM-injection in healthy and APAP-ALI mice. (F) Organ fluorescence quantification shows BMDM localisation in liver, lung, and spleen in mice receiving Vivotrack-stained BMDMs (black circles, healthy; red circles, APAP-ALI) with unstained controls (grey circles). (G). CFSE-labelled BMDMs (black arrows) were detected in liver and spleen using IHC 20 hours after i.v. injection. Scale bars, 100 µm. APAP-ALI, acetaminophen-induced acute liver injury, ATP, adenosine triphosphate; BMDMs, bone-marrow derived macrophages; CFSE, carboxyfluorescein succinimidyl ester; GFP, green fluorescent protein; i.v., intravenous.

Fig. S2

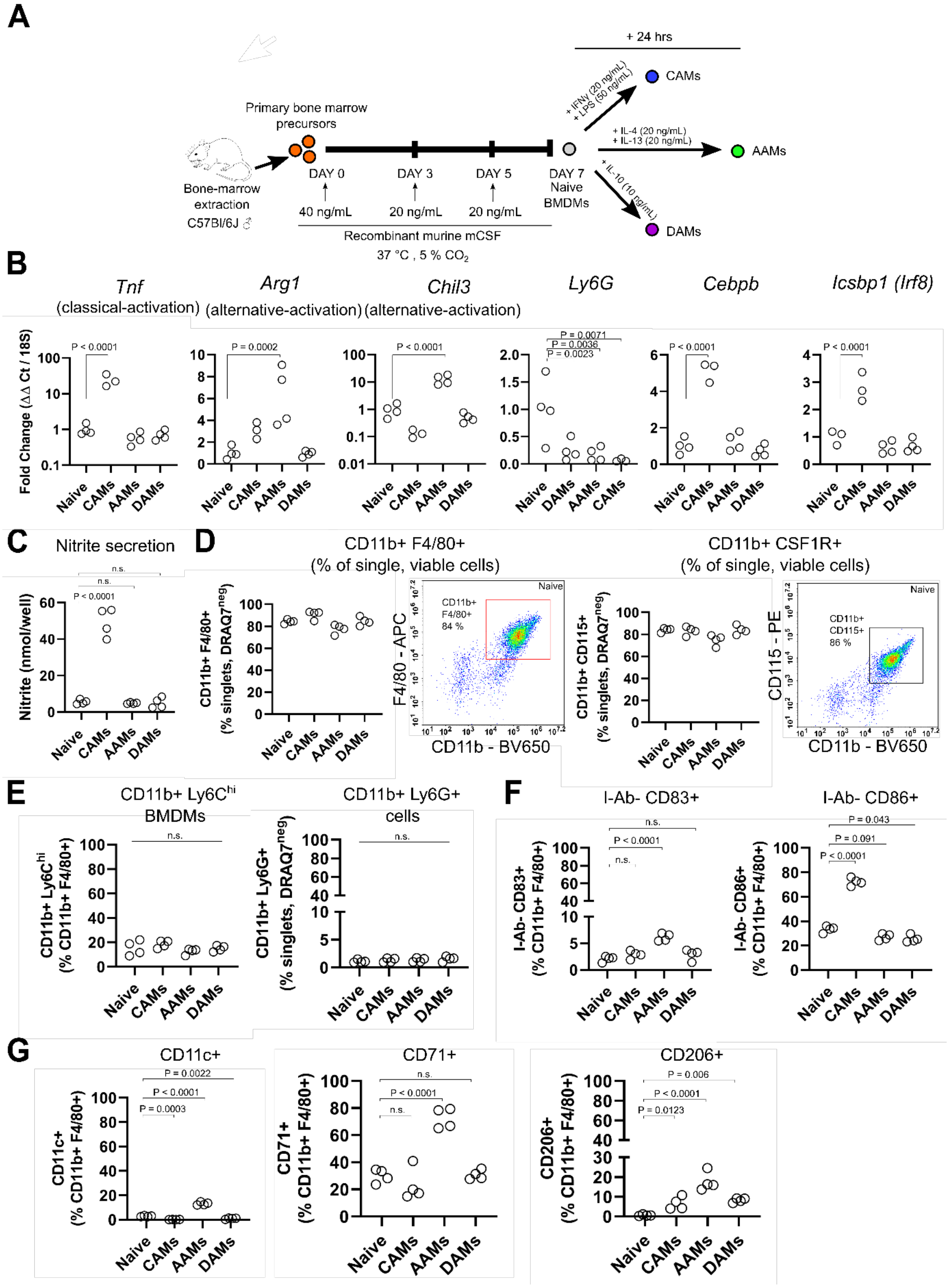

A
Primary bone marrow precursors
Bone-marrow extraction
C57Bl/6J ♂
DAY 0
DAY 3
DAY 5
DAY 7 Naive BMDMs
40 ng/mL
20 ng/mL
20 ng/mL
Recombinant murine mCSF
37 °C , 5 % CO2
+ 24 hrs
+ IFNγ (20 ng/mL) + LPS (50 ng/mL)
CAMs
+ IL-4 (20 ng/mL) + IL-13 (20 ng/mL)
AAMs
+ IL-10 (10 ng/mL)
DAMs
B
Tnf (classical-activation)
Arg1 (alternative-activation)
Chil3 (alternative-activation)
Ly6G
Cebpb
Icsbp1 (Irf8)
Fold Change (ΔΔ Ct / 18S)
P < 0.0001
P = 0.0002
P = 0.0071
P = 0.0036
P = 0.0023
Naive
CAMs
AAMs
DAMs
C
Nitrite secretion
Nitrite (nmol/well)
n.s.
D
CD11b+ F4/80+ (% of single, viable cells)
CD11b+ F4/80+ (% singlets, DRAQ7neg)
Naive
CD11b+ F4/80+ 84 %
F4/80 - APC
CD11b - BV650
CD11b+ CSF1R+ (% of single, viable cells)
CD11b+ CD115+ (% singlets, DRAQ7neg)
CD11b+ CD115+ 86 %
CD115 - PE
E
CD11b+ Ly6Chi BMDMs
CD11b+ Ly6Chi (% CD11b+ F4/80+)
CD11b+ Ly6G+ cells
CD11b+ Ly6G+ (% singlets, DRAQ7neg)
F
I-Ab- CD83+
I-Ab- CD83+ (% CD11b+ F4/80+)
I-Ab- CD86+
I-Ab- CD86+ (% CD11b+ F4/80+)
P = 0.043
P = 0.091
G
CD11c+
CD11c+ (% CD11b+ F4/80+)
P = 0.0022
P = 0.0003
CD71+
CD71+ (% CD11b+ F4/80+)
CD206+
CD206+ (% CD11b+ F4/80+)
P = 0.006
P = 0.0123

Fig. S2. Differentiation details and phenotypic characteristics of BMDM populations. (A) Schematic for differentiation protocol: Bone marrow cells (orange) were extracted from femurs and tibias of C57BL/6J males and incubated with mCSF (CSF1) for 7 days. Cultures were supplemented with CSF1 at Day 0, Day 3, and Day 5 (at indicated final concentrations) to yield mature BMDMs at day 7. BMDMs were tested or further polarised for 24 hours towards CAMs (with IFNγ/LPS), AAMs (IL-4/-13) or DAMs (IL-10) using recombinant factors at indicated final concentrations. (B) Relative gene expression quantification (determined by $2^{-\Delta\Delta CT}$ method) for indicated BMDM populations for *Tnf*, *Arg1*, *Chil3*, *Ly6g*, *Cebpb*, and *Iscbp1*. Data calibrated to naïve BMDMs after 18S rRNA normalisation (n = 3/4 biological replicates per group; see Fig. 4A for further data). (C) Secreted nitrite levels in indicated populations quantified by Greiss test (data expressed as total nmol/well to account for differences in final well volume). (D) Flow cytometry quantification with representative dot plots showing CD11b and F4/80 dual positivity (left panels), or CD11b and CSF1R dual positivity (right panels). (E) Flow cytometry quantification showing Ly6C (left panel) and Ly6G (right panel) status in BMDM preparations as indicated (see Fig. 4B for further data) (F) Flow cytometry quantification showing CD83-positive (left panel) and CD86-positive (right panel) cells in I-Ab negative populations as indicated. AAMs and CAMs showed an increase in CD83 and CD86-positivity respectively. (G) Flow cytometry quantification showing CD11c (left panel), CD71 (centre panel) and CD206 (right panel) increases in AAMs. Gates set using FMO controls. *p*-values indicated in panels, n.s., not significant. One-way ANOVA (B-G).

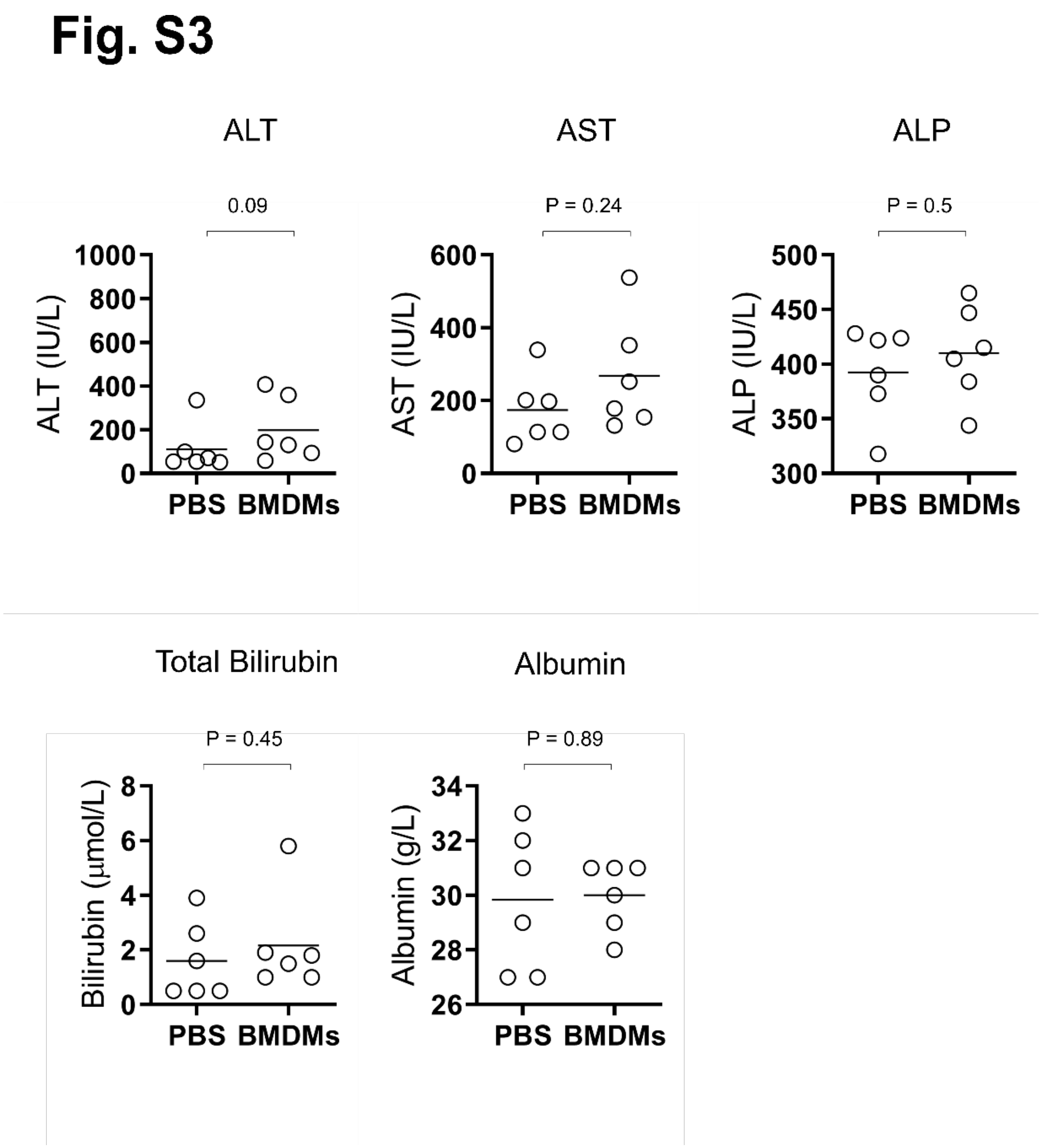

Fig. S3. BMDM injection does not change serum chemistry markers in healthy mice. Supporting details showing baseline serum liver injury or liver function markers in healthy mice after PBS or BMDM administration (n = 6 per group, 1 x 10^6, 100 µL, i.v.). Serum was obtained 20 hours post-injection and assayed for bilirubin, ALT, AST, ALP and albumin as indicated in panels. Circles represent individual mice around the mean (black line) in groups. Student's t-test or Mann-Whitney U test. *p*-values indicated in panels.

FIG. S4

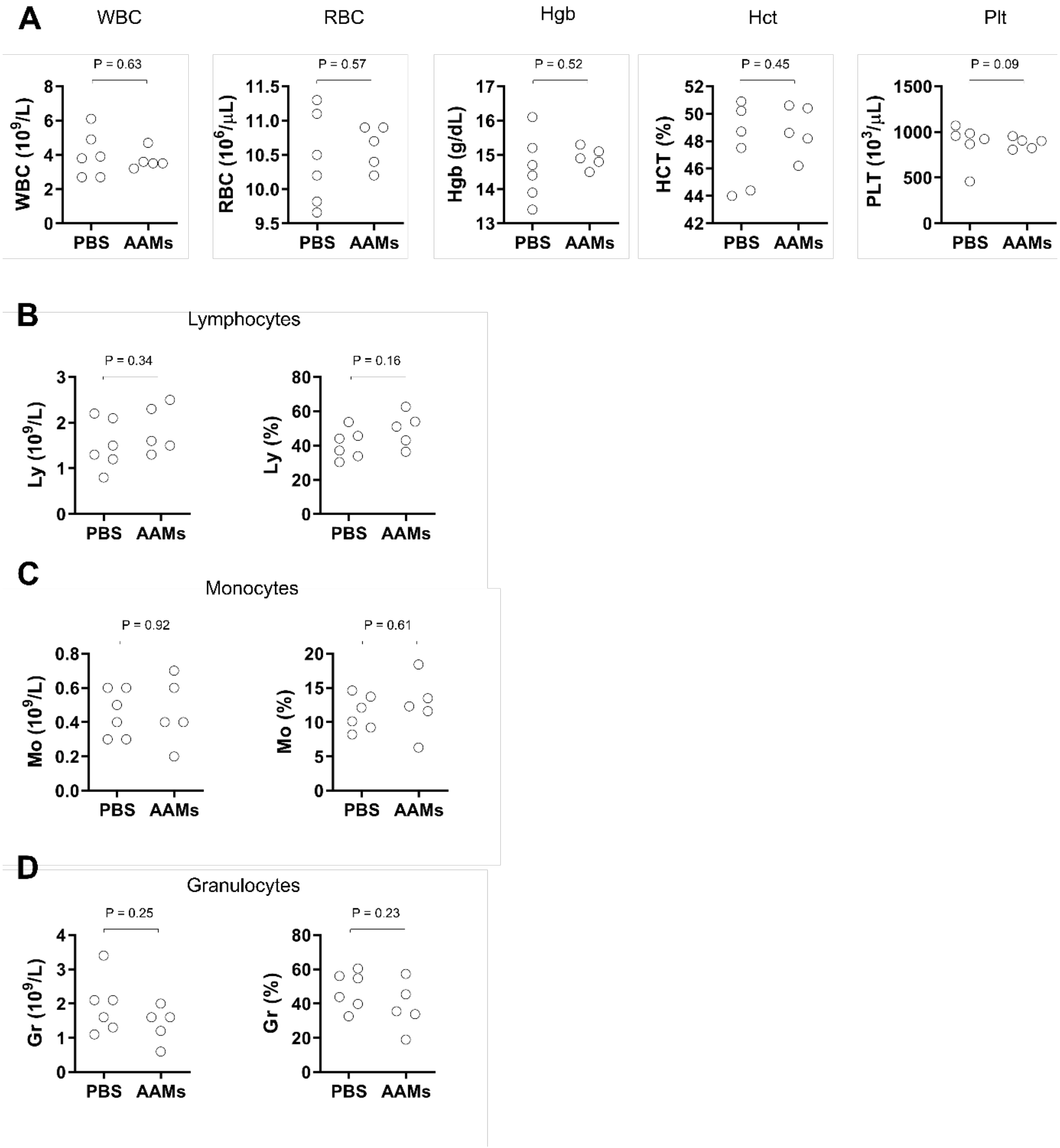

Fig. S4. BMDM injection does not change hematology markers in APAP-ALI mice. Whole blood analysis of APAP-ALI mice treated with PBS (vehicle control) or AAMs ($5x10^6$, i.v.). (A) Panels show open circles (individual animals) in groups for white blood cells (WBC), red blood cells (RBC), haemoglobin (Hgb), haematocrit (HCT), and platelets (PLT). (B) The number (left) and percentage (right) of lymphocytes per group. (C) The number (left) and percentage (right) of monocytes per group. (D) The number (left) and percentage (right) of granulocytes per group. Two-way t-test for (A) (B) (C) and (D). *p*-values indicated in panels.

Fig. S5

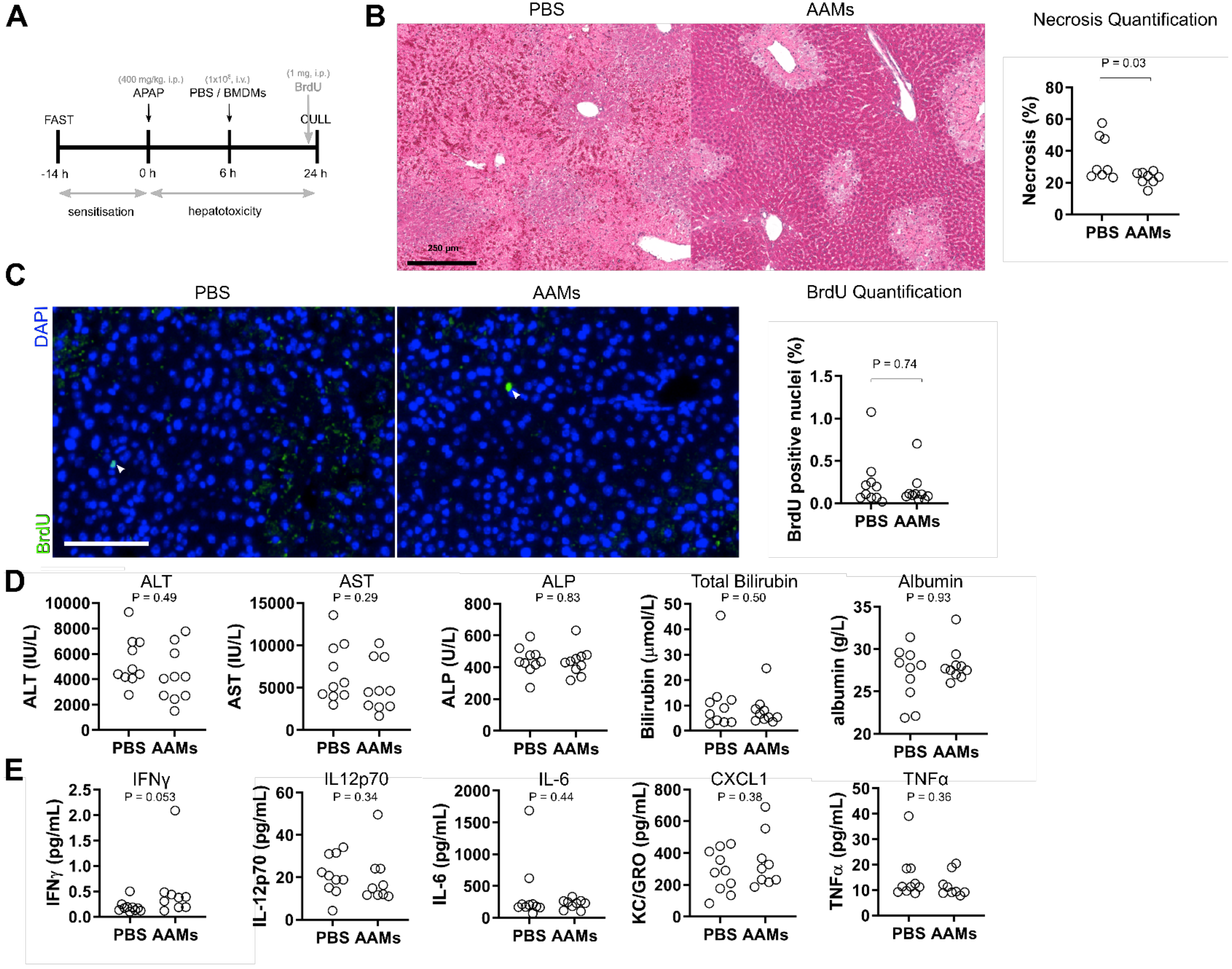
A
(400 mg/kg, i.p.)
APAP
(1x10^6, i.v.)
PBS / BMDMs
(1 mg, i.p.)
BrdU
FAST
CULL
-14 h
0 h
6 h
24 h
sensitisation
hepatotoxicity
B
PBS
AAMs
250 µm
Necrosis Quantification
P = 0.03
Necrosis (%)
PBS AAMs
C
PBS
AAMs
DAPI
BrdU
BrdU Quantification
P = 0.74
BrdU positive nuclei (%)
PBS AAMs
D
ALT
P = 0.49
ALT (IU/L)
AST
P = 0.29
AST (IU/L)
ALP
P = 0.83
ALP (U/L)
Total Bilirubin
P = 0.50
Bilirubin (µmol/L)
Albumin
P = 0.93
albumin (g/L)
E
IFNγ
P = 0.053
IFNγ (pg/mL)
IL12p70
P = 0.34
IL-12p70 (pg/mL)
IL-6
P = 0.44
IL-6 (pg/mL)
CXCL1
P = 0.38
KC/GRO (pg/mL)
TNFα
P = 0.36
TNFα (pg/mL)
PBS AAMs

Fig. S5. Delivery of AAMs at 6 hours led to reduced necrosis but did not change proliferation or serum inflammation parameters by 24 hours. (A) Study design: injection of AAMs ($1x10^6$, i.v.) or PBS vehicle alone six hours after APAP administration (400 mg/kg, i.p.) to fasted mice. Tissues and blood was harvested at 24 hours. (B) Representative H+E histological stains of liver tissue from APAP-ALI mice receiving PBS or AAMs. Right panel shows necrosis quantification (open circles represent individual mice, n ≥ 7 per group). (C) Representative IF images of BrdU incorporation in liver tissues from APAP-ALI mice treated with PBS or AAMs. BrdU incorporation was low at 24 hours in all APAP-ALI mice. (D) Serum chemistry parameters were not significant differences in APAP-ALI mice treated with PBS or AAMs. (E) Serum proinflammatory cytokines were not significantly different between PBS- and AAM-treated mice. Scale bars 100 µm. Mann-Whitney test (B) (C) (D, bilirubin and albumin only) and (E) for non-parametric datasets. Two-way t-test (D, except bilirubin and albumin) for parametric datasets. *p*-values indicated in panels.

Fig. S6

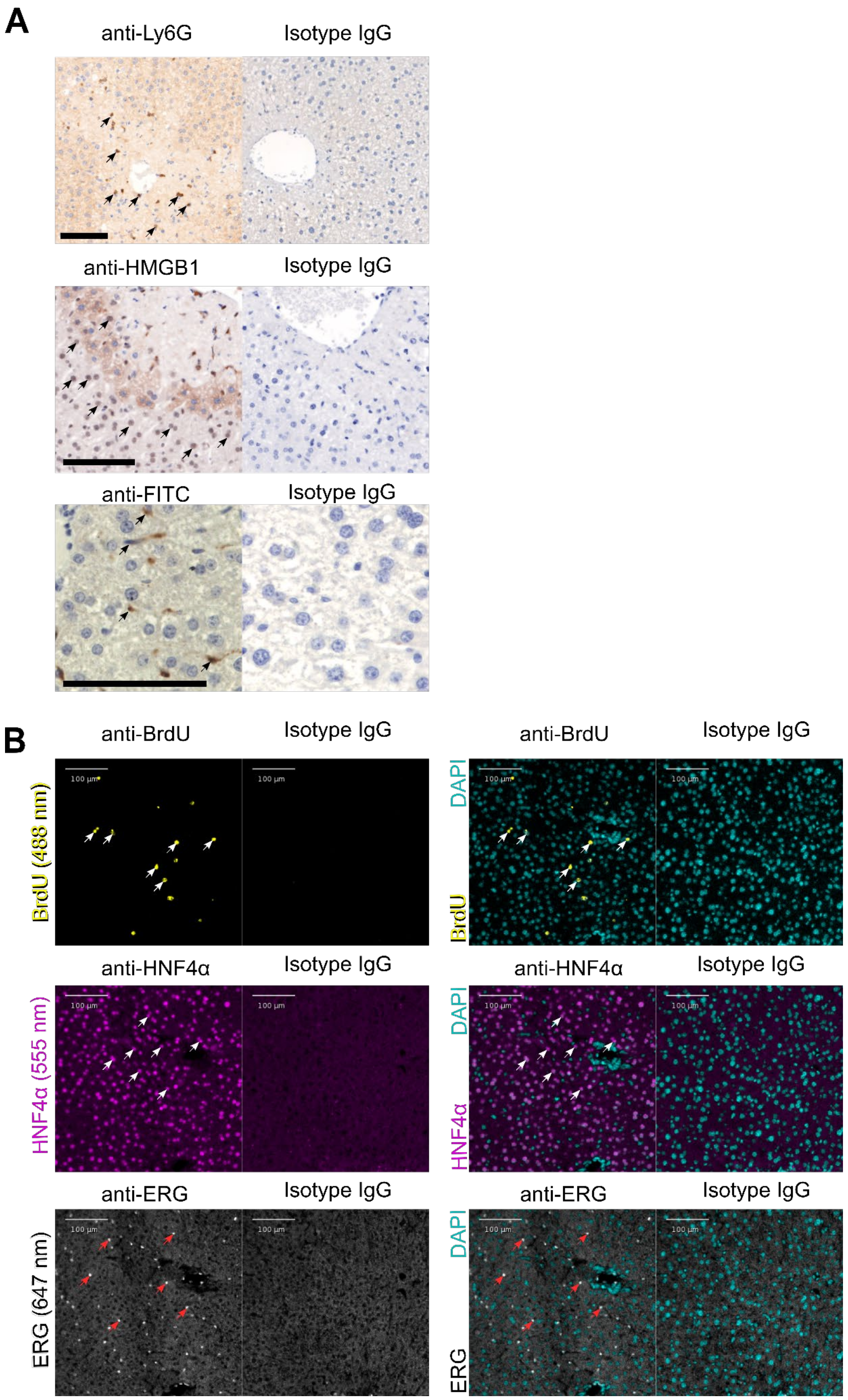

A
anti-Ly6G
Isotype IgG
anti-HMGB1
Isotype IgG
anti-FITC
Isotype IgG
B
anti-BrdU
Isotype IgG
anti-BrdU
Isotype IgG
BrdU (488 nm)
DAPI
BrdU
anti-HNF4α
Isotype IgG
anti-HNF4α
Isotype IgG
HNF4α (555 nm)
DAPI
HNF4α
anti-ERG
Isotype IgG
anti-ERG
Isotype IgG
ERG (647 nm)
DAPI
ERG

Fig. S6. Isotype controls for immunodetection assays. Supporting details showing representative immunostains for antibody and species-matched isotype IgG control antibodies. (A) Representative IHC stains show DAB-based detection (black arrows) of Ly6G (top row), HMGB1 (middle row), and FITC (bottom row) in liver tissue. Respective isotype controls in right hand panels. (B) Representative IF stains show fluorophore-based detection of BrdU (yellow nuclei, white arrows), HNF4α (magenta nuclei, white arrows), and ERG (white nuclei, red arrows). Respective isotype controls adjacent. Right hand panels show same images with DAPI overlay (cyan nuclei) with isotype controls adjacent.

Fig. S7

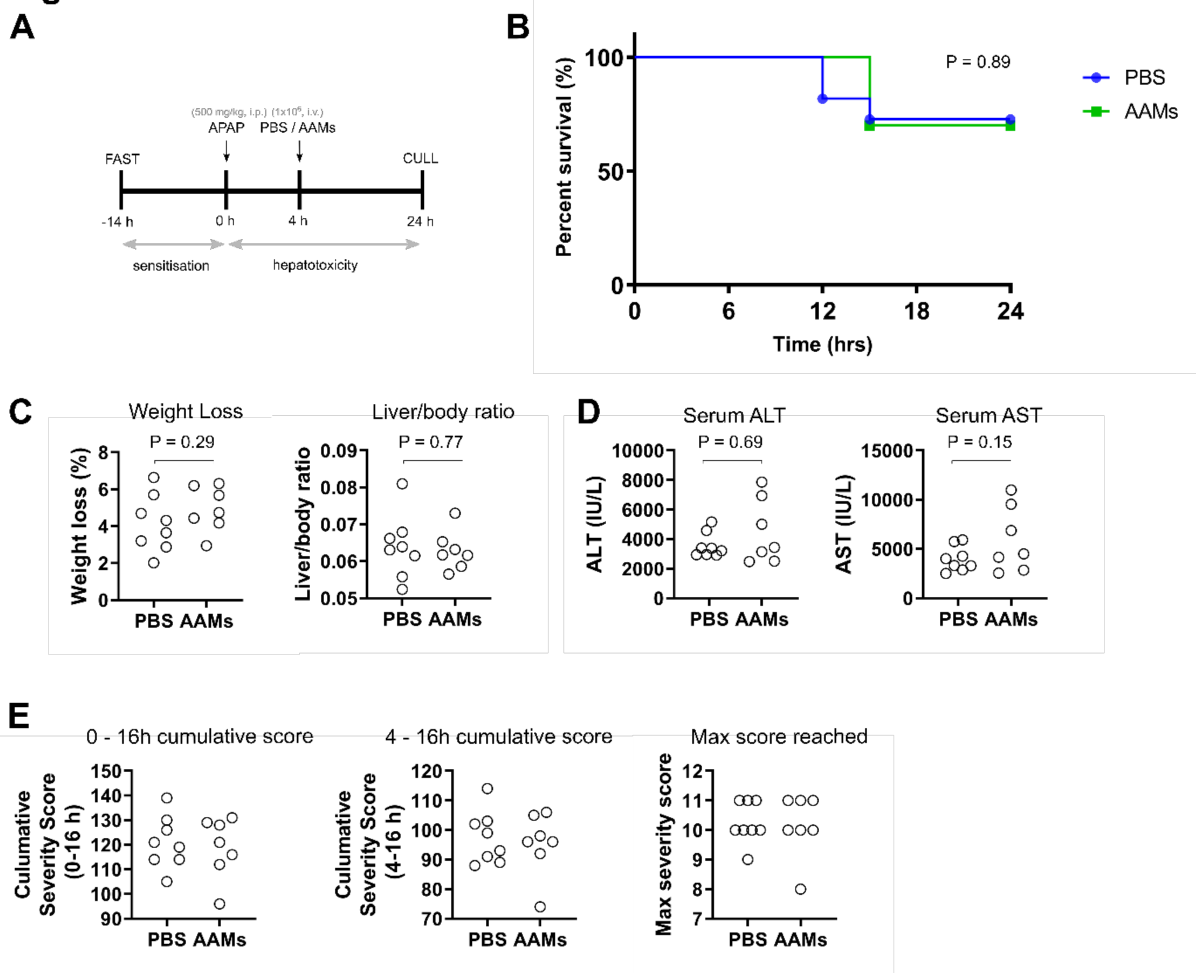

Fig. S7. AAM administration at 4 hours did not improve survival in high dose APAP-ALI mice. (A) Study design: injection of AAMs ($1x10^6$, i.v.) or PBS vehicle alone four hours after APAP administration (500 mg/kg, i.p.) to fasted mice. Tissues and blood were harvested in surviving mice at 24 hours. (B) Kaplan–Meier plot of mice that exceeded severity limits triggering humane cull. Each mouse was scored hourly (0-16 hours,) based on pre-defined parameters of APAP-ALI phenotype (refer to Table S1; n≥11, Wilcoxon test). (C) Percentage weight loss and liver/body weight ratio in APAP-ALI mice treated with PBS or AAMs. Each circle represents an individual mouse (n≥7, two-way t-test). (D) Serum transaminase activity (ALT, left; AST, right) in APAP-ALI mice treated with PBS or AAMs (n≥7, ALT, Mann-Whitney; AST, two-way t-test;). (E) Cumulative severity scores of all surviving APAP-ALI mice in each treatment group from either the entire duration (0-16 h, left panel) or post-treatment (4-16 h, middle panel). Right hand panel shows the maximum severity score reached for each mouse in each treatment group. *p*-values indicated in panels.

Fig. S8

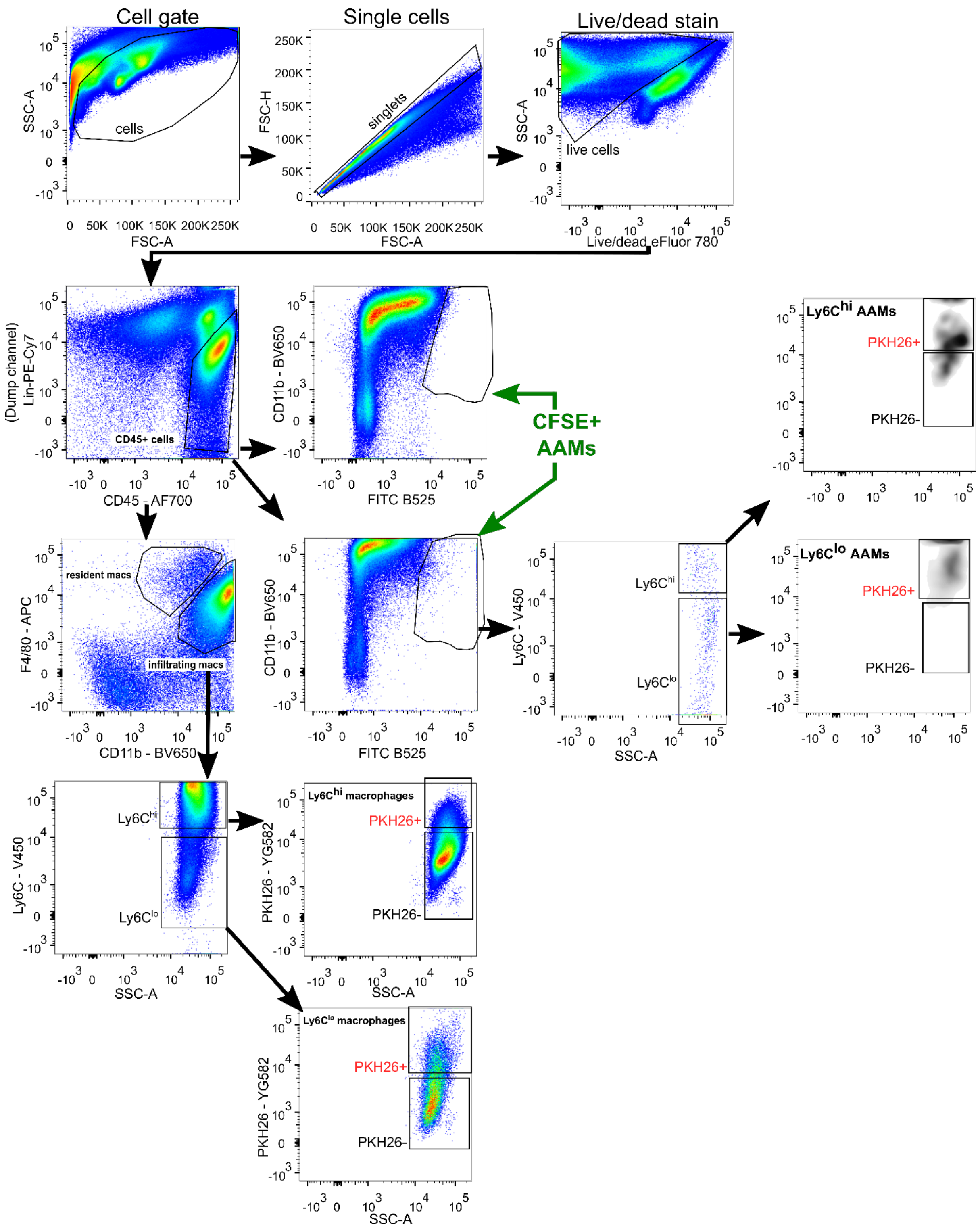

Fig S8. Gating strategy for in vivo phagocytosis labelling liver digests. Top panels show all cells used for analysis, exclusion of doublets, and selection of live cells (negative for live/dead). Sub-populations were expressed as proportions of total hepatic macrophages or CD45+cells. Hepatic resident macrophages were defined as viable CD45+ Ly6G− CD3− NK1.1− CD19− CD11b^{lo} F4/80^{hi}. Hepatic infiltrating macrophages were defined as viable CD45+ Ly6G− CD3− NK1.1− CD19− CD11b^{hi} F4/80^{lo} cells from non-parenchymal fraction of digested livers and used to identify macrophage subsets. Quantification of absolute numbers of cells per liver was performed by expressing each subset

as a proportion of NPCs, counting total number of NPCs in the digested portion of liver, calculating the total number of NPCs in the whole liver by weight differential, thereby calculating the total number of each subpopulation. Transplanted AAMs were identified as CFSE+. The percentage CFSE+ cells was calculated on the gate of total viable CD45+ Ly6G- CD3- CD19- NK1.1- cells. The negative was set on a liver from an APAP-ALI mouse receiving vehicle instead of AAMs. AAMs were further classified on their Ly6C expression, gating set using FMO (fluorescence minus one) controls. The percentage of phagocytic cells (positive and negative) was calculated on the gate of CFSE+ cells. The negative was set using the liver from an APAP-ALI mouse transplanted with AAMs but injected with the vehicle instead of PKH26PCL. PKH26PCL MFI was calculated in the same gate.

Fig. S9

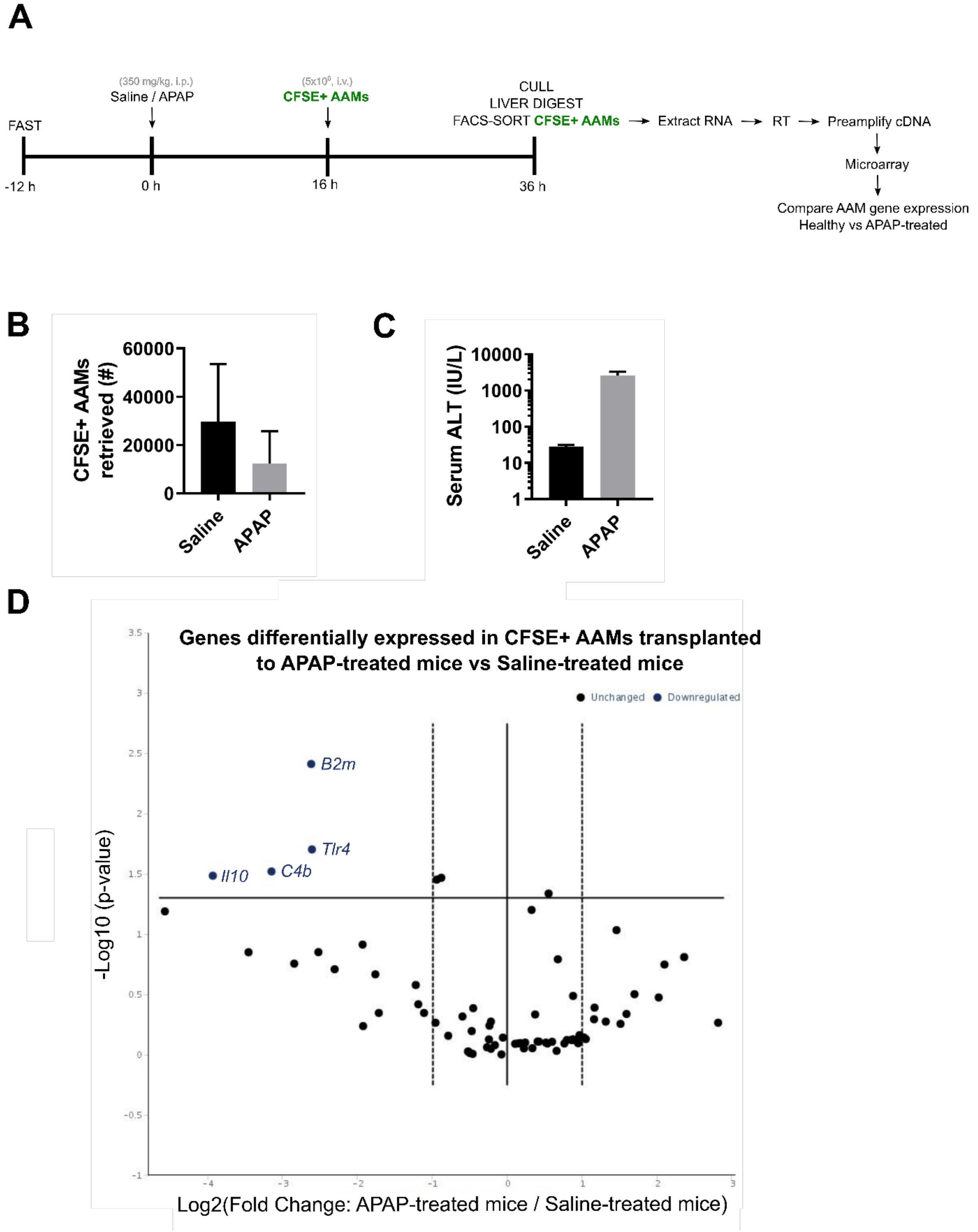

Fig. S9. AAMs largely retain their phenotype post-injection in APAP-ALI mice. (A) CFSE-AAMs ($5x10^6$) were injected intravenously into either healthy or APAP-ALI mice at 16 hours before cull at 36 hours. Liver tissue was digested to isolate myeloid cells, and exogenous AAMs FACS-sorted for downstream PCR array analysis. (B) Graph shows mean (± SD) of number of isolated viable CFSE+ AAMs following tissue digest and FACS-sorting in healthy (saline) and APAP-ALI mice (n=3, biological replicates). (C) Serum ALT activity in healthy (saline) and APAP-ALI mice. (D) Volcano plot showing differentially expressed genes in AAMs injected into APAP-ALI mice versus AAMs

injected into healthy mice. The x-axis indicates fold-change (Log2 scale) of each gene, and the y-axis indicates p-values (-Log10 scale). The horizontal line represents the threshold for significance, and the vertical dashed line indicate the threshold for statistically-relevant fold-changes. Four genes in the dataset (*B2m*, *Tlr4*, *C4b*, and *Il10*) were significantly downregulated (upper left quadrant) (n=3, two-way t-test). *Actb*, *Gusb*, and *Hsp90ab1* served as housekeeping genes between healthy and APAP-ALI mice using the arithmetic mean.

Fig. S10

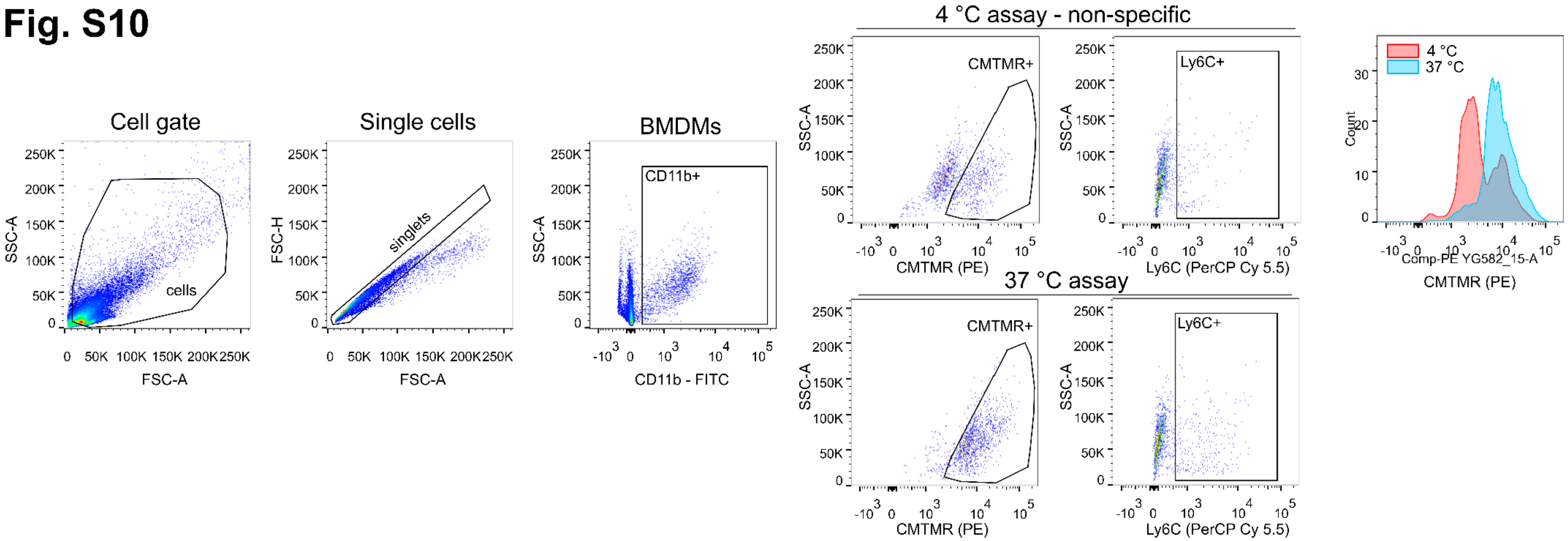

Fig. S10. Gating strategy for in vitro phagocytosis experiments. Supporting details showing gating strategy for categorising cell sub-populations for in vitro phagocytosis analysis**.** Panels show all cells analysed in the study, excluding doublets. BMDMs were identified as CD11b+. The percentage of phagocytic cells was calculated on the gate of CD11b+ cells. BMDMs positive for phagocytosis were defined as CMTMR+ (i.e. fluorescent apoptotic thymocytes). The percentage of non-specific binding (phagocytosis at 4 °C, top right panels) was subtracted from the percentage of phagocytic BMDMs (at 37 °C). Phagocytic cells were further classified based on their Ly6C expression (bottom right panels). Gates were set using FMO controls

Fig. S11

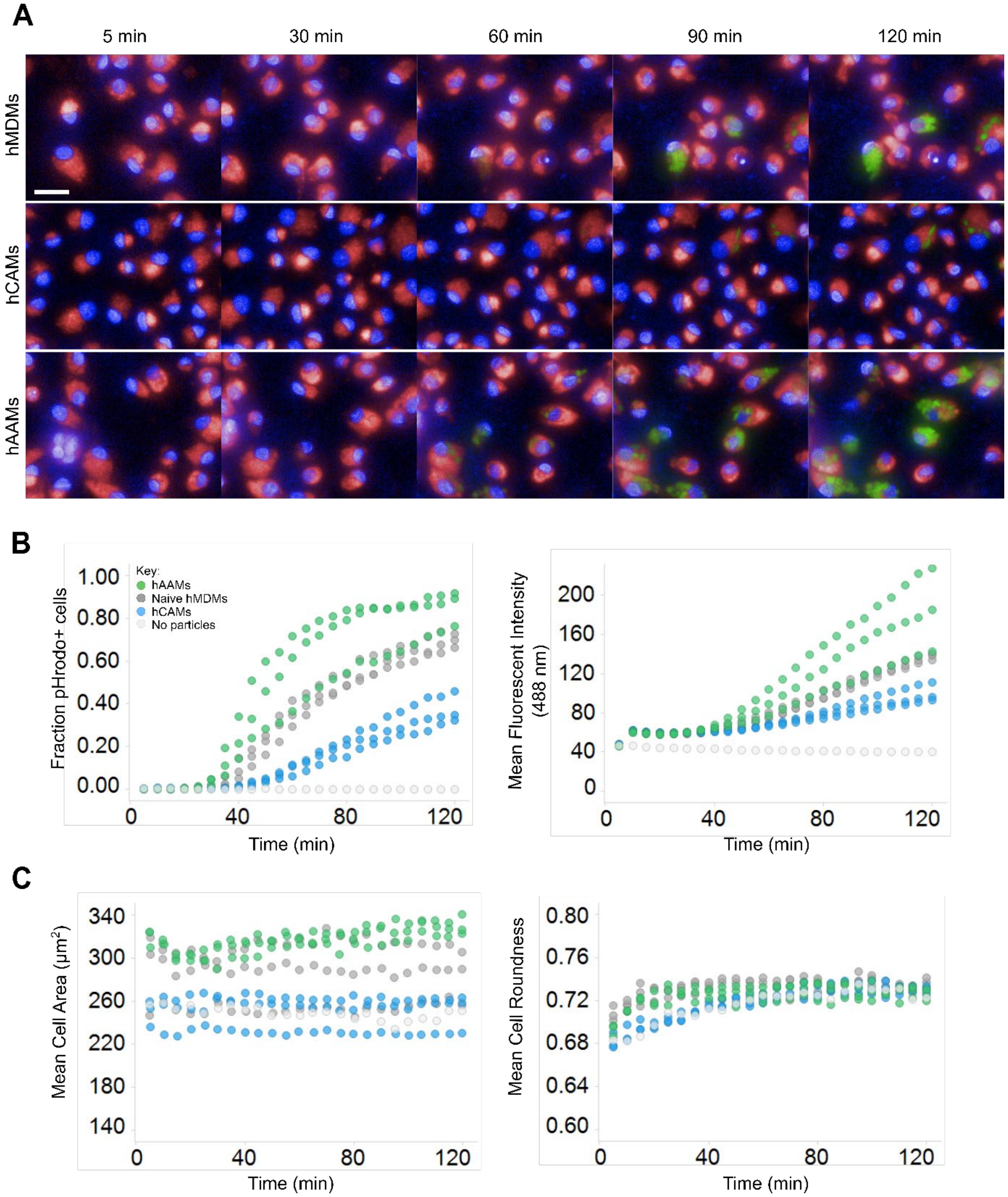

Fig. S11. Human AAMs are highly phagocytic in vitro. (A) Representative images during a real-time phagocytosis assay taken at indicated times. Human naïve MDMs (top row), hCAMs (middle row) and hAAMs (bottom row) are shown (Deep Red CellMask, red; NucBlue, blue). Phagocytosis of zymosan-bioparticles is indicated by increases in intracellular fluorescence (green). (B) Phagocytosis quantification expressed as the fraction of pHrodo-positive cells (left) and MFI of total cells in hMDM populations (right). (C) Morphological analysis of hMDM populations during phagocytosis, cell size (left) and roundness (right). Scale bars 20 µm. AAMs, alternatively-activated macrophages; BMDMs, bone-marrow derived macrophages; CAMs, classically-activated macrophages; MFI, mean fluorescent intensity.

Fig. S12

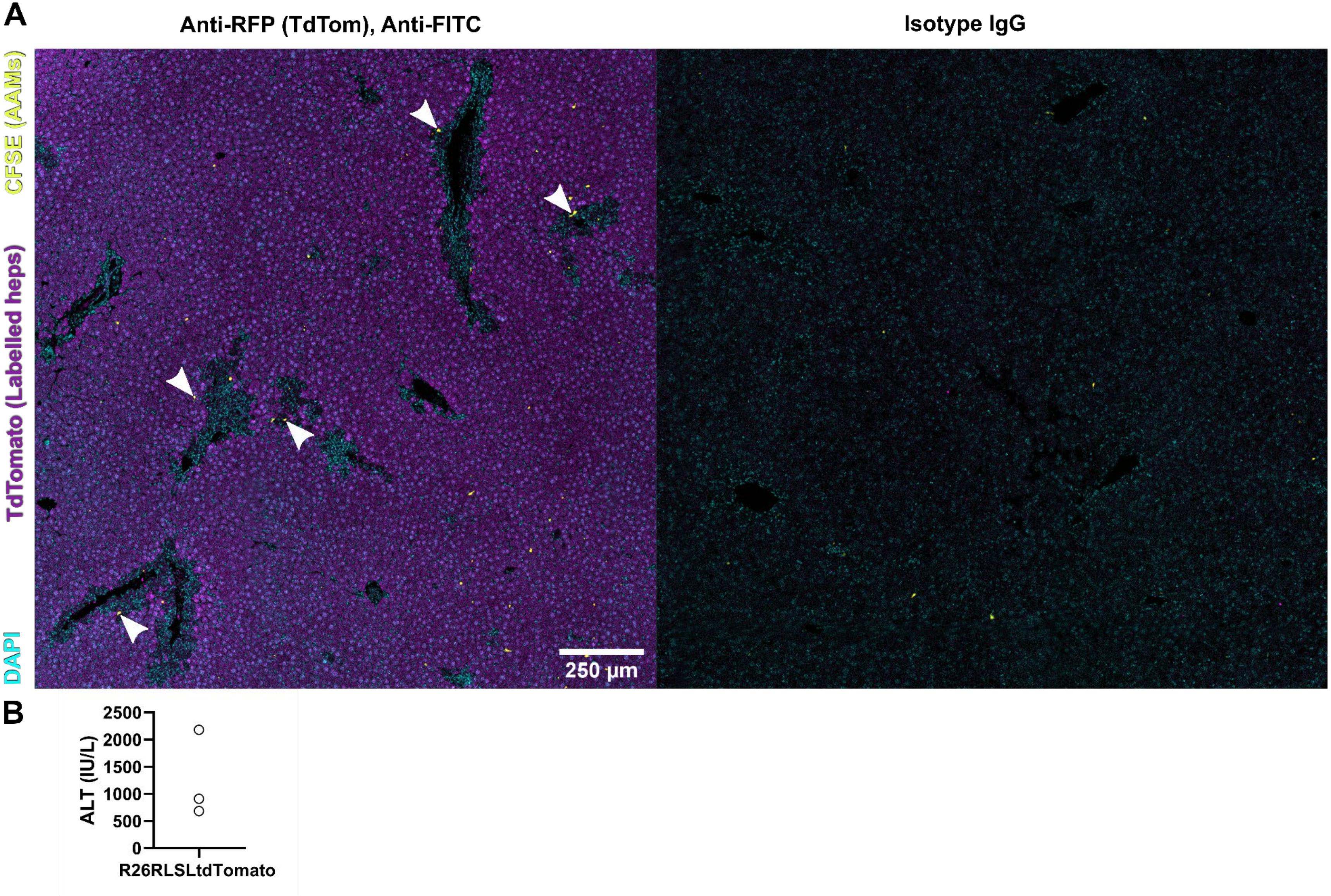
A
Anti-RFP (TdTom), Anti-FITC
Isotype IgG
CFSE (AAMs)
TdTomato (Labelled heps)
DAPI
250 µm
B
2500
2000
1500
1000
500
0
ALT (IU/L)
R26RLSLtdTomato

Fig. S12. CFSE-labelled AAMs localise throughout liver parenchyma including peri-necrotic regions in TdTomato-labelled APAP-ALI mice. (A) AAV8.Tbg-Cre injection (5x10^{11} virus particles, i.v.) was performed in R26RLSL tdTomato mice to express TdTomato specifically in hepatocytes. IF was performed to boost TdTomato and FITC fluorescence using anti-RFP and anti-FITC antibodies before confocal microscopy. Panel shows representative IF images (left, antibody treated; right, isotype control) showing injected AAMs (yellow, white arrowheads) throughout the parenchyma and TdTomato+ hepatocytes (magenta) against DAPI counterstain (cyan). Centrilobular areas of hepatocyte necrosis contained TdTomato-negative infiltrating cells with punctate areas of TdTomato debris. (B) Serum ALT activity in R26LSLTdTomato mice 36 hours after APAP administration. Circles represent individual mice.

Fig. S13

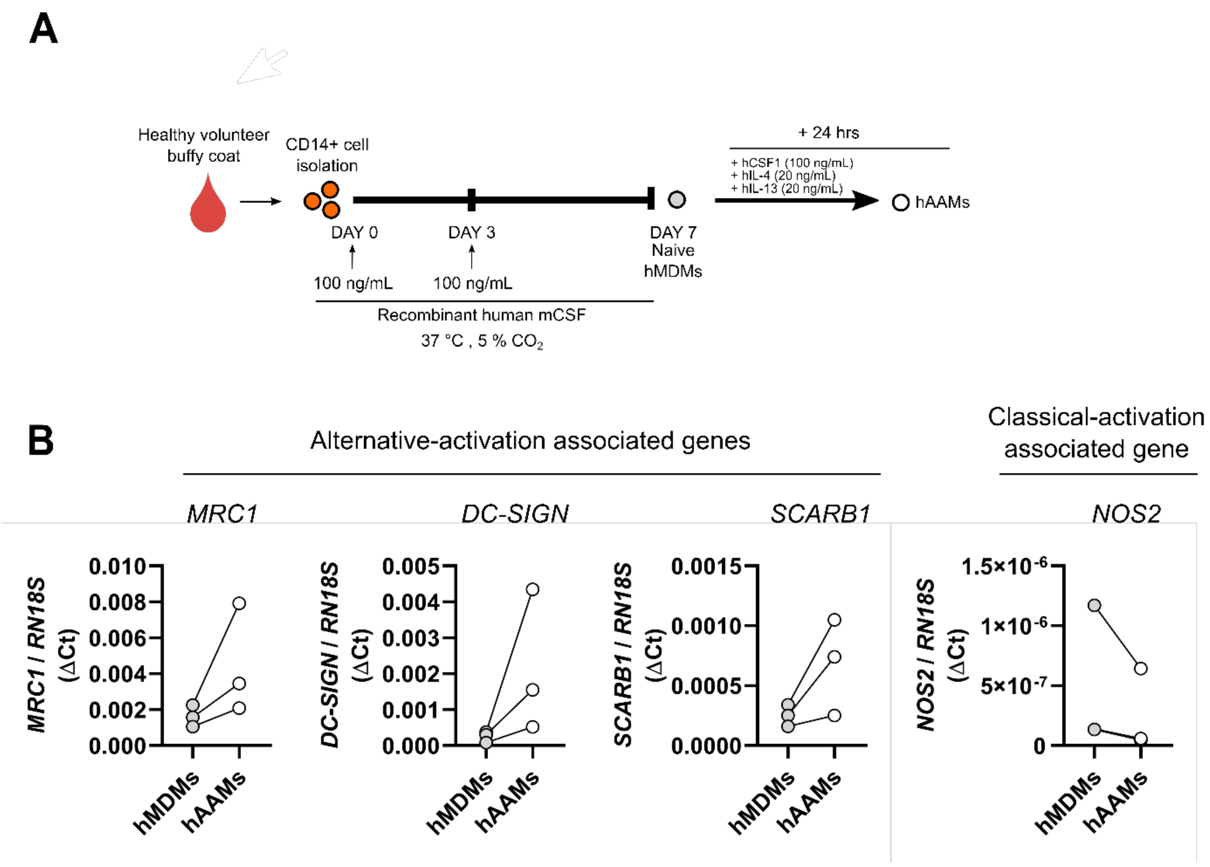

Fig. S13. hAAM generation and gene expression analysis. (A) Schematic for differentiation protocol: CD14+ cells (orange circles) were extracted from buffy coats derived from healthy volunteer blood donation. Cultures were supplemented with hCSF1 at Day 0 and Day 3 (at indicated final concentrations) to yield mature hMDMs at day 7 (grey circles). hMDMs were further polarised for 24 hours towards hAAMs (white circles) using recombinant factors. (B) Gene expression analysis of indicated genes (determined by $2^{\Delta CT}$ method) in individual-matched hMDM (grey circles) and hAAMs (white circles), black lines connect samples derived from the same donor.

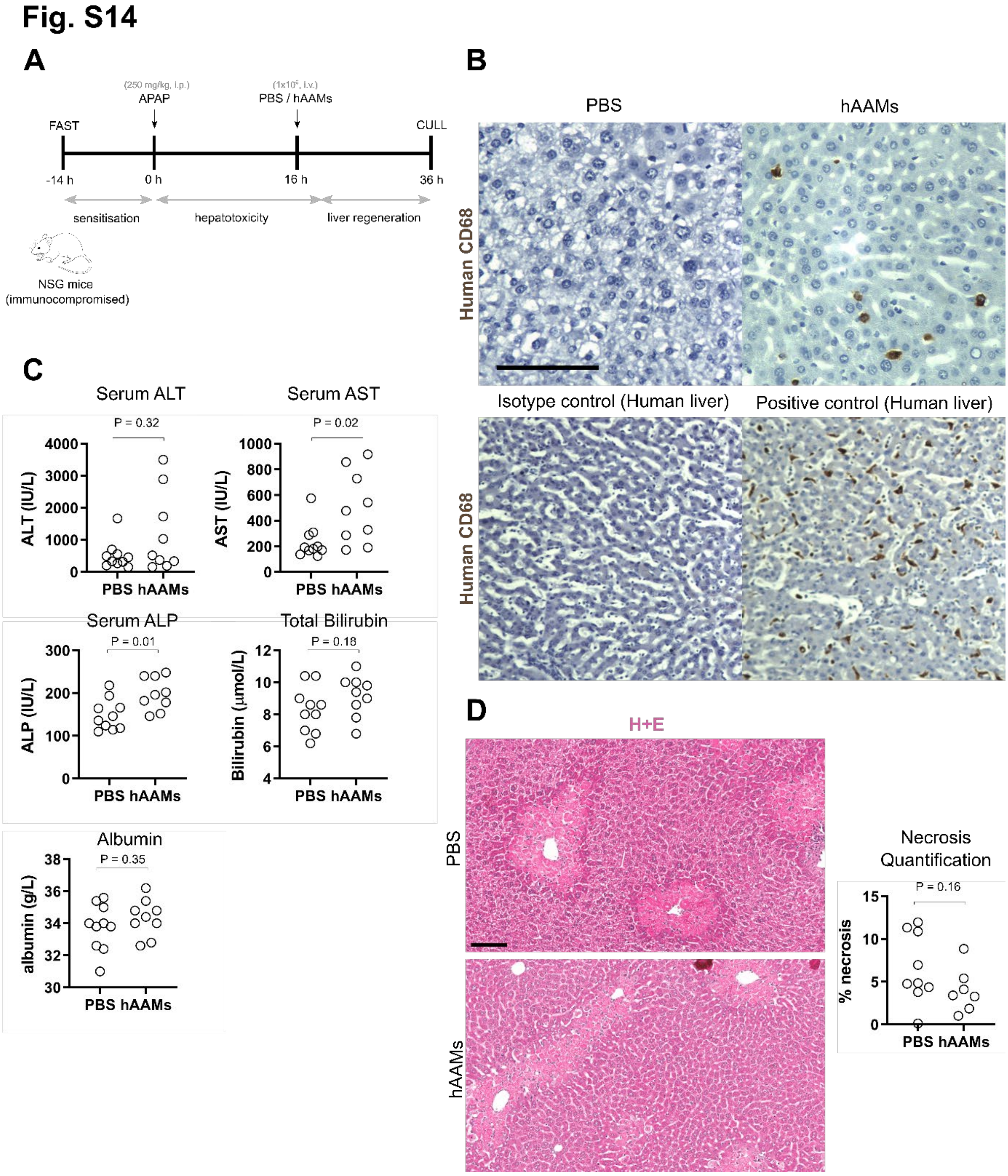

Fig. S14. hAAM injection was not efficacious in immunocompromised APAP-ALI mice (A) Experimental design: injection of hAAMs (1x10^6, i.v.) or PBS vehicle alone sixteen hours after APAP-injection in fasted NSG mice. Mice were humanely culled at 36 hours. (B) Representative DAB-based IHC stains using anti-CD68 primary antibody on fixed liver sections. Top panels show PBS- and AAM-injected mice (left, and right respectively). Bottom panels show IHC on fixed human liver tissue (isotype IgG control, left; anti-CD68, right). (C) Serum chemistry values (ALT, AST, Bilirubin, ALP, and albumin, as indicated in the panels) of APAP-ALI mice treated with PBS or hAAMs in groups. Each circle represents an individual mouse. (D) Representative images of H+E stains showing centrilobular necrotic lesions in APAP-ALI mice treated with PBS (left image) or hAAMs (right image). Right hand graph provides quantification of necrotic tissue as a percentage of total tissue. Scale bars 100 µm. Two-way t-test or Mann-Whitney test for parametric and non-parametric datasets respectively in (C). *p*-values indicated in panels.

Fig. S15

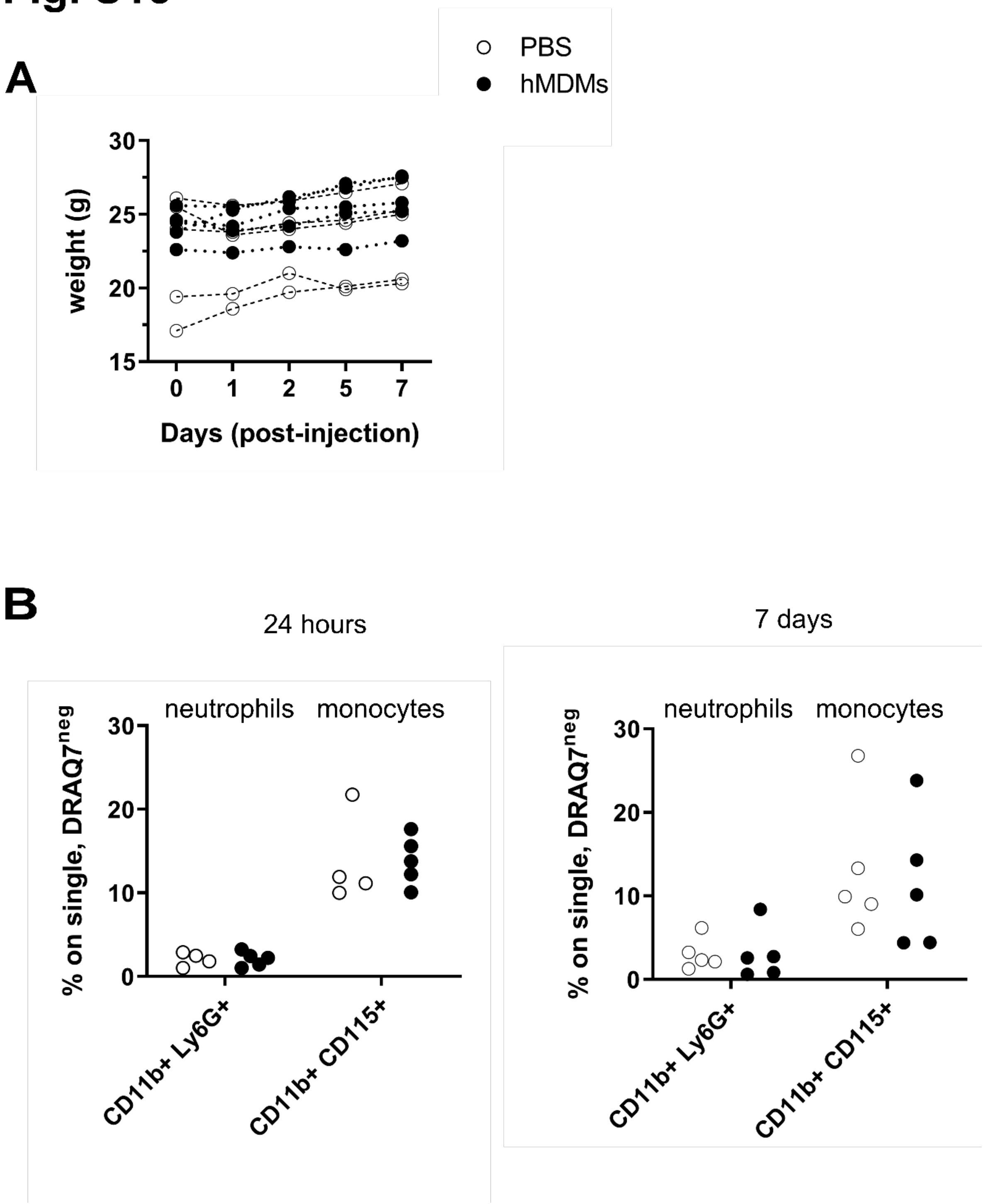

Fig. S15. hAAM transfer is well tolerated in immunocompetent WT mice. (A) Panel shows individual body weights of healthy C57BL6/J mice treated with PBS (open circles) or hMDMs (black circles, $1x10^6$, i.v.). Dashed line connects repeated measurements from the same mice. (B) Percentage of blood neutrophils (CD11b+ Ly6G+) and monocytes (CD11b+ CD115+) in healthy C57BL6/J mice treated with PBS (open circles) or hMDMs (black circles). Blood neutrophils and monocytes were analysed at 24 hours (left panel) and 7 days (right panel) post-injection.

Table S1

Hunching		Score	Skin (paw/ear) paleness		Score
Not hunched		0	Absent		0
Mild hunching		1	Mild pallor		1
Moderate hunching		2	Moderate pallor		2
Very hunched		3	Very pale		3
**Responsiveness to touch**			**Neurological symptoms**		
Normal		0	Normal		0
Responds to interaction but slow		1	Slow walk		1
Response delayed and slow		2	Impaired ability to walk straight		2
Unresponsive		3	Lethargic, immobile,		3
			no recovery from the supine position		
			when placed by user		
**Piloerection**			**Breathing**		
Not present		0	Normal		0
Mild piloerection		1	Mild respiratory effort		1
Moderate piloerection		2	Moderate respiratory effort		2
Very pronounced piloerection		3	Very laboured breathing		3

Table S1. APAP phenotypic scoring sheet. Each mouse was scored (0-3) hourly on each parameter after APAP administration. To prevent mice exceeding severity limits in order to comply with Home Office regulations, the following pre-defined thresholds were applied: within the first six hours, if mice score 8 or above, or score 3 for either 'neurological symptoms' or 'responsiveness to touch', then proceed with humane cull. After six hours, if mice score 12 or above, or score 3 for either 'neurological symptoms' or 'responsiveness to touch', then proceed with humane cull.

Table S2

Gene Symbol	AVG ΔC_t		2^(-ΔCt)		Fold Change		Fold Up- or Down-Regulation
	APAP	PBS	APAP	PBS		*P*-Value	APAP/PBS
Bcl6	8.51	8	0.00274	0.00391	0.7	0.948	-1.43
C3	3.82	5.85	0.07059	0.01736	4.07	0.334	4.07
C3ar1	4.83	3.12	0.03505	0.11494	0.3	0.460	-3.28
C4b	8.27	5.13	0.00323	0.0286	0.11	0.030	-8.86
Ccl1	8.51	9.39	0.00274	0.00149	1.84	0.751	1.84
Ccl11	8.51	8.29	0.00274	0.00319	0.86	0.532	-1.16
Ccl12	8.51	8.29	0.00274	0.00319	0.86	0.892	-1.16
Ccl17	8.51	7.4	0.00274	0.00592	0.46	0.452	-2.16
Ccl19	8.51	8.06	0.00274	0.00375	0.73	0.411	-1.37
Ccl2	2.86	4.02	0.13795	0.06145	2.24	0.406	2.24
Ccl20	8.51	9.39	0.00274	0.00149	1.84	0.751	1.84
Ccl22	8.51	9.39	0.00274	0.00149	1.84	0.751	1.84
Ccl24	8.51	6.75	0.00274	0.00928	0.3	0.217	-3.39
Ccl25	8.51	7.99	0.00274	0.00394	0.7	0.940	-1.44
Ccl3	-0.45	0.15	1.36184	0.89848	1.52	0.780	1.52
Ccl4	-0.54	-0.1	1.4495	1.08089	1.34	0.779	1.34
Ccl5	7.15	2.58	0.00702	0.16673	0.04	0.064	-23.75
Ccl7	2.14	4.96	0.22671	0.03218	7.05	0.534	7.05
Ccl8	8.51	9.39	0.00274	0.00149	1.84	0.752	1.84
Ccr1	7.69	9.39	0.00484	0.00149	3.25	0.314	3.25
Ccr2	7.8	9.39	0.00449	0.00149	3.02	0.458	3.02
Ccr3	8.51	8.05	0.00274	0.00377	0.73	0.986	-1.38
Ccr4	8.51	9.39	0.00274	0.00149	1.84	0.752	1.84
Ccr7	5.62	5.96	0.02027	0.01605	1.26	0.879	1.26
Cd14	5.4	3.48	0.02361	0.08955	0.26	0.591	-3.79
Cd40	8.51	7.72	0.00274	0.00473	0.58	0.699	-1.73
Cd40lg	8.51	9.39	0.00274	0.00149	1.84	0.752	1.84
Cebpb	-0.04	-0.3	1.02495	1.21326	0.84	0.746	-1.18
Crp	8.51	9.39	0.00274	0.00149	1.84	0.752	1.84
Csf1	7.27	8.15	0.00646	0.00351	1.84	0.325	1.84
Cxcl1	8.51	9.39	0.00274	0.00149	1.84	0.752	1.84
Cxcl10	7.49	4.97	0.00556	0.03188	0.17	0.141	-5.74
Cxcl11	8.51	9.39	0.00274	0.00149	1.84	0.752	1.84
Cxcl2	0.44	0.97	0.73827	0.50894	1.45	0.810	1.45
Cxcl3	8.36	9.31	0.00304	0.00158	1.93	0.803	1.93
Cxcl5	8.51	9.39	0.00274	0.00149	1.84	0.752	1.84
Cxcl9	8.51	9.39	0.00274	0.00149	1.84	0.752	1.84
Cxcr1	8.51	9.39	0.00274	0.00149	1.84	0.752	1.84
Cxcr2	8.51	9.39	0.00274	0.00149	1.84	0.752	1.84
Cxcr4	7.59	6.37	0.00517	0.01205	0.43	0.268	-2.33
Fasl	8.51	9.39	0.00274	0.00149	1.84	0.752	1.84
Fos	-0.2	-1.1	1.14781	2.20211	0.52	0.037	-1.92
Ifng	5.44	6.96	0.02297	0.00805	2.85	0.554	2.85
Il10	7.28	3.35	0.00643	0.098	0.07	0.033	-15.24
Il10rb	3.36	3.3	0.09732	0.10122	0.96	0.720	-1.04
Il17a	8.51	9.39	0.00274	0.00149	1.84	0.752	1.84
Il18	5.04	3.11	0.03044	0.116	0.26	0.123	-3.81
Il1a	7.29	6.82	0.00639	0.00886	0.72	0.635	-1.39
Il1b	3.87	1.56	0.0685	0.33811	0.2	0.196	-4.94
Il1r1	8.51	9.39	0.00274	0.00149	1.84	0.752	1.84
Il1rap	7.32	7.69	0.00625	0.00483	1.3	0.462	1.3
Il1rn	4.59	6.69	0.04139	0.00966	4.29	0.179	4.29
Il22	8.51	9.39	0.00274	0.00149	1.84	0.752	1.84
Il23a	8.51	9.03	0.00274	0.00192	1.43	0.794	1.43
Il23r	8.51	7.32	0.00274	0.00624	0.44	0.381	-2.28
Il5	8.51	9.39	0.00274	0.00149	1.84	0.752	1.84
Il6	8.51	8.66	0.00274	0.00248	1.11	0.805	1.11
Il6ra	3.51	4.67	0.08791	0.03934	2.23	0.505	2.23
Il7	8.51	9.31	0.00274	0.00158	1.74	0.754	1.74
Il9	8.51	9.39	0.00274	0.00149	1.84	0.752	1.84
Itgb2	0.49	2.85	0.71148	0.13827	5.15	0.150	5.15

Kng1	8.51	9.39	0.00274	0.00149	1.84	0.752	1.84
Lta	8.51	9.39	0.00274	0.00149	1.84	0.752	1.84
Ltb	8.51	8.68	0.00274	0.00243	1.13	0.801	1.13
Ly96	4.31	1.47	0.05038	0.36154	0.14	0.176	-7.18
Myd88	8.51	8.75	0.00274	0.00232	1.18	0.792	1.18
Nfkb1	4.67	4.44	0.03916	0.04614	0.85	0.579	-1.18
Nos2	8.51	9.39	0.00274	0.00149	1.84	0.752	1.84
Nr3c1	8.51	5.06	0.00274	0.03002	0.09	0.142	-10.95
Ptgs2	8.51	9.27	0.00274	0.00162	1.7	0.806	1.7
Ripk2	8.51	8.24	0.00274	0.00331	0.83	0.872	-1.21
Sele	8.51	9.39	0.00274	0.00149	1.84	0.752	1.84
Tirap	7.22	7.05	0.00672	0.00752	0.89	0.837	-1.12
Tlr1	8.51	8.62	0.00274	0.00255	1.08	0.811	1.08
Tlr2	4.79	5.02	0.03604	0.03087	1.17	0.883	1.17
Tlr3	8.51	8.01	0.00274	0.00387	0.71	0.970	-1.41
Tlr4	4.3	1.69	0.05084	0.30969	0.16	0.019	-6.09
Tlr5	8.51	7.55	0.00274	0.00532	0.52	0.544	-1.94
Tlr6	8.51	9.37	0.00274	0.00151	1.81	0.752	1.81
Tlr7	8.51	8.92	0.00274	0.00207	1.33	0.776	1.33
Tlr9	8.51	7.91	0.00274	0.00415	0.66	0.482	-1.51
Tnf	7.54	7.47	0.00536	0.00565	0.95	0.988	-1.05
Tnfsf14	8.51	9.39	0.00274	0.00149	1.84	0.752	1.84
Tollip	1.74	2.42	0.29914	0.18714	1.6	0.157	1.6
Actb	-2.63	-2.3	6.18549	4.93217	1.25	0.069	1.25
B2m	3.12	0.5	0.11494	0.70493	0.16	0.004	-6.13
Gapdh	-0.01	1.45	1.00618	0.36574	2.75	0.093	2.75
Gusb	1.5	2.06	0.35247	0.24019	1.47	0.045	1.47
Hsp90ab1	1.12	0.24	0.45868	0.84414	0.54	0.035	-1.84

Table S2. Low-density PCR array data for FACS-sorted AAMs after injection into APAP-ALI or healthy mice (n=3 biological replicates per group). Average delta Ct to the mean value of the housekeeping genes (AVG ΔCt) indicated per group, transformed average delta Ct 2^(-ΔCt) indicated per group, the fold change, *p*-value, and fold-change (negative value indicates a downregulation compared to PBS control group).

Table S3

Antigen	Supplier	Catalogue #	Species	Protein conc. (mg/mL)	Dilution	Antigen retrieval
FITC	ThermoFisher	71-1900	Rabbit	0.25	1:200	15 min, sodium citrate (pH 6.0)<br><br>15 min TE buffer (pH 9.0)
BrdU	Abcam	ab6326	Rat	1.00	1:200	15 min, sodium citrate (pH 6.0),<br><br>15 min TE buffer (pH 9.0)
HMGB1	Abcam	Ab18256	Rabbit	1.0 (varies)	1:500	10 min TE buffer, 5 min pre-heat (pH 9.0)
HNF4α	Perseus Proteomics	PP-1415	Mouse	0.10	1:200	15 min TE buffer (pH 9.0)
Ly6G	Biolegend	127602	Rat	0.50	1:1000	10 min, sodium citrate (pH 6.0)
ERG	Abcam	ab92513	Rabbit	0.88	1:125	15 min, sodium citrate (pH 6.0)
mCherry (recognises tdTomato)	SICGEN	AB0081-500	Goat	3.00	1:400	15 min TE buffer (pH 9.0)
CD68	Abcam	ab213363	Rabbit	0.71	1:8000	10 min TE buffer, 5 min pre-heat (pH 9.0)

Table S3. Primary antibody conditions used in immunodetection assays.

Table S4

Gene Name	Quantitect Assay #
Csf1	QT01164324
Tgfb1	QT00145250
Il6	QT00098875
Cxcl1	QT00115647
Ccl5	QT01747165
Tnf	QT00104006
Arg1	QT00134288
Chil3	QT01549156
Retnla	QT00254359
Ly6g	QT00529655
Cebpb	QT00320313
Icsbp	QT00174195
Nos2	QT00100275
Il10	QT00106169
Ly6c	QT00247604

Table S4. Primer details used in qPCR assays.

Supplementary methods

Serum chemistry. Serum was isolated from whole blood after centrifugation (14,000 g) after clotting. Serum chemistry was performed by measurement of alanine aminotransferase (ALT), aspartate aminotransferase (AST), alkaline phosphatase (ALP), total bilirubin, and serum albumin. ALT was measured using a described method,[1] utilising a commercial kit (Alpha Laboratories Ltd). AST and ALP were determined by a commercial kit (Randox Laboratories). Total bilirubin was determined by the acid diazo method described by Pearlman and Lee[2] using a commercial kit (Alpha Laboratories Ltd). Mouse serum albumin measurements were determined using a commercial serum albumin kit (Alpha Laboratories Ltd). All kits were adapted for use on a Cobas Fara centrifugal analyser (Roche Diagnostics Ltd). For all assays, intra-run precision was CV < 4%. In some experiments, assays were run on plasma samples.

Plasma microRNA measurement. RNA was isolated from 10 µL once-frozen plasma using miRNeasy serum/plasma kit (Qiagen) following the manufacturer's protocol. cDNA was prepared from 5 µL purified RNA using miScript II kit (Qiagen) with HiSpec buffers according to the manufacturer's protocol. cDNA was diluted 1:10 in RNase-free water before performing PCR in duplicate on a Roche Lightcycler 480 in 384-well format using miScript SYBR Green PCR Kit (Qiagen) using primer assays for murine miR-122 and following the manufacturer's protocol. Relative expression was determined using the $2^{-\Delta\Delta CT}$ method using the mean healthy Ct value obtained from each mouse (pre-APAP), with reference to let-7d, which served as housekeeping miRNA as used previously.[3]

Immunodetection assays. Four micron sections of formalin-fixed paraffin-embedded (FFPE) blocks were collected on Superfrost™ Plus slides. Sections were dewaxed and rehydrated before heat-induced antigen retrieval. Sections were blocked in protein solution (Spring Bioscience) for 30 min, before overnight incubation with primary antibodies (see

Supplementary table 3 for details) at 4 °C, or isotype-control IgG (Vector Laboratories). For immunofluorescence, sections were washed extensively with PBS before application of secondary antibody: Alexa Fluor® 488, 555, or 647 conjugate for 60 mins in a dark environment. Washed sections were stained with DAPI (1 μg/mL, 15 min) before mounting in Fluoromount-G (ThermoFisher). For IF-based quantification, twenty non-overlapping regions were imaged on Operetta high content imaging system (PerkinElmer) using a 10x objective. Intensity of fluorescent signal was quantified in DAPI-positive nuclei, and thresholds applied using isotype controls to identify positive cells. For immunohistochemical stains, a biotinylated secondary antibody was incubated (1:500) for 1 hour before treatment with avidin-based peroxidase reagent (Vector Laboratories). Targets were visualised using DAB colorimetric development using a Harris' haematoxylin counterstain. Sections were washed in acid alcohol and Scott's tap water substitute, dehydrated and mounted with ClearVue mountant (Thermo Fisher) before imaging with a Vectra® Polaris™ imaging system (PerkinElmer). Total positive cells/per 200x field were quantified using Image-J Fiji. A minimal of eight individual fields were analysed per sample.

Serum and liver cytokines. Cytokines were quantified in serum or liver homogenate using a V-plex Proinflammatory Panel kit (Meso Scale Discovery) following the manufacturer's instructions. Serum (25 μL) or liver homogenate (100 μg total protein, in 12.5 μL) was diluted in the plate using buffers provided. Liver tissue was homogenized in lysis buffer (150 mM NaCl, 20 mM Tris, 1 mM EDTA, 1 mM EGTA, 1 % Triton X-100, 2 x protease inhibitor cocktail, (Sigma Aldrich)). Total protein was determined using BCA protein assay kit (Thermo Fisher). Plate was read on a QuickPlex SQ 120 analyzer (Meso Scale Discovery). Standards were assayed in duplicate and samples in singlet as recommended.

Haematoxylin and eosin staining and necrosis quantification. Four micron sections were stained with haematoxylin and eosin (H+E) on a Shandon Varistain Gemini ES Automated Slide Stainer (ThermoScientific) and mounted using the Shandon ClearVue Coverslipper (ThermoScientific). For necrosis quantification, two methods were used at different times during the project. First method: H+E slides were scanned to create a single image with Dotslide VS-ASW software (Olympus) using a motorized stage and an Olympus BX51 microscope, acquiring images using an Olympus PlanApo 2X lens and Olympus XC10 camera. Images were analysed using the Trainable WEKA Segmentation plugin[4] in FIJI[5, 6]. A separate classifier identifying necrotic tissue, healthy tissue and vasculature, was determined for each sample. Second method: H+E sections were scanned on a Vectra Polaris Automated Quantitative Pathology Imaging System (Perkin Elmer) using multispectral imaging. At least six 10x ROIs (representing a minimum combined tissue area of 15.5 mm^2) were analysed for each sample. An algorithm was built to identify healthy tissue, necrotic tissue, and vasculature after training using a representative image from every sample, and then batch processed on all images. For both methods, necrosis was quantified by expressing necrotic tissue area as a percentage of total tissue area.

Greiss test. Nitrite levels were quantified in BMDM supernatants using Griess Reagent Kit (G-7921, Invitrogen, via ThermoFisher Scientific) following the manufacturer's instructions in a flat-bottomed 96-well microplate. Absorbance was read at 548 nm and data extrapolated form the standard curve.

Gene expression analysis. RNA was harvested from cells using the RNeasy Mini Kit (Qiagen) according to the manufacturer's instructions. RNA was quantified using Nanodrop ND-1000 (Thermo Scientific) and reverse transcribed using Quantitect Reverse Transcription kit

(Qiagen) including genomic DNA removal step. Gene expression was quantified using QuantiTect SYBR Green PCR Kit (Qiagen) in 384-well format on a Roche Lightcycler 480 II (Roche). For proinflammatory gene expression analysis on liver tissue from APAP-treated mice, approx. 10 mg frozen liver tissue was weighed and homogenized in 900 µL Qiazol lysis reagent (Qiagen) using the tissue tearor. Chloroform was mixed and incubated (5 min) before centrifugation (12000 g, 15 min, 4 °C). Aqueous phase was separated and RNA precipitated with propan-2-ol. After an ethanol wash, RNA was resuspended from air-dried pellets using RNAse free water. For gene expression, Quantitect primers were used (Qiagen, see table S4). Housekeeping genes were *Rn18s* for cells and *Gapdh* for tissue. Relative expression was determined using the $2^{-\Delta\Delta CT}$ method versus biological controls with reference to the housekeeping genes.

RT^2 PCR array. CFSE-stained AAMs were collected from digested livers of healthy and APAP-ALI mice as above, except collected directly into lysis buffer (Qiagen) after sorting via FACS (FACS ARIA II, BD Biosciences). Lysates were homogenized using QIAshredder (Qiagen) columns following manufacturer's instructions. Total RNA was obtained using RNeasy Micro Kit (Qiagen), before pre-amplification using RT^2 PreAMP cDNA Synthesis Kit (Qiagen). Amplified cDNA was assayed using a catalogued RT^2 profiler PCR array (077ZG, Qiagen) with a Roche Lightcycler 480 II PCR machine (384-well format, Roche). Data was analyzed using GeneGlobe online portal (Qiagen, http://www.qiagen.com/geneglobe). All samples passed data quality control criteria. The C_T cut-off was set to 35. Normalisation was performed using arithmetic mean of *Actb*, *Gusb*, and *Hsp90ab1.* Volcano plots were generated by plotting fold-change ($2^{-\Delta Ct}$) versus P-value (calculated based on a Student's t-test of n = 3 replicates). The treatment and control groups were AAMs from APAP-treated and PBS-treated mice respectively. Dataset provided in table S2.

Phagocytosis assays. Unpolarized BMDMs (naïve), CAMs, or AAMs were used to investigate phagocytosis. BMDMs were incubated with apoptotic thymocytes (primary thymocytes harvested from 3-5 week old C57BL6 mice, treated with 1 μM hydrocortisone, as previous[7]. Apoptotic thymocytes were labelled using CMTMR (Invitrogen) according to the manufactuer's instructions[8]. BMDMs were challenged with labelled apoptotic cells for 30 min, 1 hour, or 2 hours at a 1:5 ratio at 37 °C or 4°C. Cells were washed and phagocytosis verified by flow cytometry after staining (1:200) with anti-CD11b FITC (clone M1/70; eBioscience) and anti-Ly6C PerCP/Cy5.5 (clone HK1.4; eBioscience). Phagocytosis was calculated as percentage of CD11b+ CMTMR+ cells at 37 °C minus percentage of CD11b+ CMTMR+ cells at 4 °C. Percentage of Ly6C+ cells was calculated in the gate of CD11b+ CMTMR+ cells at 37 °C. Mean fluorescence intensity (MFI) for CMTMR was calculated on the same gate in the same conditions. Data were acquired on a LSRII Fortessa (BD Biosciences). Data analysis and graphical interpretation performed with FlowJo X software (FlowJo LLC).

For real time experiments, mouse BMDMs or hMDMs were plated ($1x10^5$/well) in 96-well CellCarrier microplates (PerkinElmer) overnight before stimulation with appropriate cytokines (see methods above) to drive polarisation. Before imaging, BMDMs were stained with NucBlue live cell stain (ThermoFisher) and CellMask Deep Red plasma membrane stain (ThermoFisher) according to the manufacturer's instructions. Plates were transferred to Operetta high-content imaging system (PerkinElmer) and allowed to equilibrate at 37 °C and 5 % CO_2. Phagocytosis was initiated by the addition of pHrodo green zymosan bioparticles (ThermoFisher) to the wells following manufacturer's instructions. Fluorescent images were taken in the DAPI channel, 488 nm, and 647 nm before, and at 5 min intervals after the addition of bioparticles for a maximum of 150 min. Images were quantified on Columbus image analysis

software (PerkinElmer). Macrophages positive for phagocytosis were classified based on a fluorescence intensity (488 nm) greater than 500 and expressed as a fraction of all live cells (NucBlue positive cells). Mean fraction values were taken from four separate wells per group.

Liver digest, leukocyte isolation, and flow cytometry. To examine the localisation and phenotype of transplanted AAMs *in vivo*, CFSE-stained AAMs or vehicle was transplanted to mice at 16 hours after APAP administration. Three hours later, mice were injected with PKH26PCL (100 µL, 0.1 mM, i.v.) to label phagocytic cells as described[9]. At 36 hours, blood was collected from sacrificed mice in EDTA-tubes and processed immediately on a Celltac α analyzer (Nihon Kohden) for hematological analysis. Plasma was harvested from the remainder of the blood via centrifugation (3800 g, 10 min, 4 °C) and frozen. Liver was digested following methods previously described[10, 11] with minor modifications. Briefly, livers were perfused with PBS and the left lateral lobe was collected at 36 hours post-APAP administration in ice-cold RPMI media. Lobes were mechanically disrupted with a scalpel in 5 mL liver digestion enzyme cocktail (Collagenase V, Sigma, 0.8 mg/mL; Collagenase D, Roche, 0.63 mg/mL; Dispase, Gibco, 1 mg/mL; DNase 1, Roche, 100 µg/mL). Homogenates were digested for 25 min in a shaking incubator at 37 °C at 240 rpm. Liver digests were passed through a 70 µm filter and made up to 30 mL with RPMI. Digests were centrifuged (300 g, 5 min, 4 °C) and washed before red blood cell lysis treatment (Sigma). Cells were counted and stained using a panel of antibodies to target cell surface markers with appropriate controls (i.e. unstained, fluorescence minus one). Non-specific antibody binding was blocked by incubating cells with 10 % mouse serum for 20 min at 4 °C followed by incubation with combinations of primary antibodies (1:200 dilution unless otherwise stated) for 20 min at 4 °C. The following conjugated antibodies were used: CD11b BV650 (clone M1/70; eBioscience), Ly-6C V450 (clone HK1.4; eBioscience), CD45.2 AF700 (clone 104; eBioscience), F4/80 APC (1:100, clone BM8;

Invitrogen), Ly-6G PE-Cy7 (clone 1A8; BioLegend), CD3 PE-Cy7 (clone 17A2; BioLegend), NK1.1 PE-Cy7 (clone PK136; BioLegend), CD19 PE-Cy7 (clone 6D5; BioLegend), CD31 PE-Cy7 (clone 390, eBioscience). Cell viability was assessed with Fixable Viability Dye eFluor780 (1:1000, eBioscience) according to manufacturer's protocols. After antibody staining, samples were either analyzed immediately or fixed with BD Cell Fix (BD Bioscience) before analysis on an LSRII Fortessa (BD Biosciences) flow cytometer. Data were analyzed using FlowJo10 software (Tree Star). For gating strategy of target populations, see Fig. S6 and Fig. S7.

Flow cytometry for human macrophages. Flow cytometry was performed on a MACSQuant VYB flow cytometer. Briefly, cell products were washed, and resuspended in PBS-EDTA (2.5 mM), containing 0.5 % Alburex® (CSL Behring). Cells were blocked with human FcR blocking reagent (Miltenyi Biotec) for 20 min, 4 °C before incubation with conjugated antibodies. Unstained controls were collected before viability assessment through DRAQ7 (Abcam) exclusion. The following antibody panels were performed sequentially in the presence of DRAQ7: 1. Release Criteria Panel: CD45 VioBlue (clone: 5B1, Miltenyi Biotec), CD14 PE (clone TÜK4, Miltenyi Biotec), CD206 FITC (clone: DCN228, Miltenyi Biotec), 25F9 APC (clone: 25F9, eBioscience). 2. Extended Panel 1: CD14 VioBlue (TÜK4, Miltenyi Biotec), CCR2 PE (clone: K036C2, BioLegend), CD163 FITC (clone: GHI/61.1, Miltenyi Biotec), CD169 APC (clone: 7-239, BioLegend). 3. Extended Panel 2: CD14 VioBlue (TÜK4, Miltenyi Biotec), CD86 PE (clone: BU63, BioLegend), HLA FITC (clone: Tu39, BioLegend). All antibodies were incubated at 1:200 dilution (20 min, 4 °C), except H (1:400), before centrifugation (300 g, 5 min) and washing in PE-A.

Hepatocyte labelling and confocal microscopy. R26RtdTomatoLSL male mice (Jackson laboratory) were administered AAV8-Tbg-Cre ($2.5x10^{11}$, i.v., Addgene) to induce TdTomato expression specifically in hepatocytes. Mice were allowed a two week washout period before APAP administration and murine CFSE-labelled AAM delivery 16 hours later. Immunofluorescent staining for mCherry (recognises tdTomato) and FITC was performed on FFPE liver sections to improve signal detection. Confocal images were obtained on a Leica SP8 confocal microscope using the navigator function in LASX software. Z-stack images were recorded at 40X magnification and presented as a single slice or a maximum projection over 7 slices representing 2.4 µm.

In vivo cell tracking. BMDMs were stained *in vitro* with VivoTrack 680 (near infra-red fluorescent imaging agent, Perkin Elmer) prior to transplantation. BMDMs ($5x10^{6}$) were incubated with reconstituted VivoTrack (2 mL) in 4 mL DPBS and incubated for 15 min at room temperature in a dark environment and subsequently washed before injection (i.v) to control or APAP-ALI mice at 16 hours. Anaesthetized mice were maintained with gaseous oxygen/isofluorane had abdominal fur clipped before fluorescent images were taken in the dorsal position at 15 min intervals using 687 nm excitation and 722 nm emission (background correction at 487 nm) on a PhotonIMAGER™ (Biospace Lab) imaging suite. After 4 hours mice were humanely culled and organs imaged *ex vivo* to confirm BMDM localisation. In some experiments, BMDMs differentiated from constitutively-expressing GFP cells[12] were used to confirm staining with Vivotrack 680 *in vitro*.

Supplementary methods references

[1] Bergmeyer HU, Scheibe P, Wahlefeld AW. Optimization of methods for aspartate aminotransferase and alanine aminotransferase. Clin Chem 1978;24:58-73.
[2] Pearlman FC, Lee RT. Detection and measurement of total bilirubin in serum, with use of surfactants as solubilizing agents. Clin Chem 1974;20:447-453.
[3] Lewis PS, Dear J, Platt V, Moggs J, Goldring C, Park BK. Reply. Hepatology 2012;55:1642.
[4] Frank E, Hall M, Trigg L, Holmes G, Witten IH. Data mining in bioinformatics using Weka. Bioinformatics 2004;20:2479-2481.
[5] Schindelin J, Arganda-Carreras I, Frise E, Kaynig V, Longair M, Pietzsch T, et al. Fiji: an open-source platform for biological-image analysis. Nat Methods 2012;9:676-682.
[6] Schneider CA, Rasband WS, Eliceiri KW. NIH Image to ImageJ: 25 years of image analysis. Nat Methods 2012;9:671-675.
[7] Ferenbach DA, Ramdas V, Spencer N, Marson L, Anegon I, Hughes J, et al. Macrophages expressing heme oxygenase-1 improve renal function in ischemia/reperfusion injury. Mol Ther 2010;18:1706-1713.
[8] Bosurgi L, Brunelli S, Rigamonti E, Monno A, Manfredi AA, Rovere-Querini P. Vessel-associated myogenic precursors control macrophage activation and clearance of apoptotic cells. Clin Exp Immunol 2015;179:62-67.
[9] Campana L, Starkey Lewis PJ, Pellicoro A, Aucott RL, Man J, O'Duibhir E, et al. The STAT3-IL-10-IL-6 Pathway Is a Novel Regulator of Macrophage Efferocytosis and Phenotypic Conversion in Sterile Liver Injury. J Immunol 2018;200:1169-1187.
[10] Ramachandran P, Pellicoro A, Vernon MA, Boulter L, Aucott RL, Ali A, et al. Differential Ly-6C expression identifies the recruited macrophage phenotype, which orchestrates the regression of murine liver fibrosis. Proc Natl Acad Sci U S A 2012;109:E3186-3195.
[11] Bain CC, Hawley CA, Garner H, Scott CL, Schridde A, Steers NJ, et al. Long-lived self-renewing bone marrow-derived macrophages displace embryo-derived cells to inhabit adult serous cavities. Nat Commun 2016;7:ncomms11852.
[12] Gilchrist DS, Ure J, Hook L, Medvinsky A. Labeling of hematopoietic stem and progenitor cells in novel activatable EGFP reporter mice. Genesis 2003;36:168-176.
